# Empirical analysis of the correlation between China’s Macroeconomic Market and Crude Oil Market based on mixed-frequency group factor model

**DOI:** 10.1371/journal.pone.0336227

**Published:** 2026-01-28

**Authors:** Jiaxin Zhao, Junping Yin

**Affiliations:** 1 Northeast Normal University, Academy for Advanced Interdisciplinary Studies, Changchun, China; 2 Institute of Applied Physics and Computational Mathematics, Beijing, China; 3 Shanghai Zhangjiang Institute of Mathematics, Shanghai, China; Shanghai Jiao Tong University, CHINA

## Abstract

This paper examines the asymmetric correlation and dynamic interaction between China’s macroeconomic market and the global crude oil market, addressing a critical limitation in existing literature: the frequency mismatch between high-frequency (daily) crude oil data and low-frequency (monthly) macroeconomic data. To resolve this, we employ a mixed-frequency group factor model that decomposes volatility drivers into two mutually exclusive components: (1) common factors, which capture cross-market spillovers between the two markets; and (2) group-specific factors, including low-frequency (LF)-specific factors (for China’s macroeconomic indicators) and high-frequency (HF)-specific factors (for crude oil prices). Our empirical analysis uses a comprehensive dataset spanning January 2005 to March 2024, covering 11 daily crude oil price indicators and 60 monthly Chinese macroeconomic indicators. We validate results using the adjusted coefficient of determination (*R*^2^) and Bayesian Information Criterion (BIC) for model selection, and further test robustness across three samples: a full sample (2005.01–2024.03) and two crisis sub-samples (2007.01–2009.12 Financial Crisis, 2020.01–2023.12 COVID-19). Three core findings emerge: First, the two markets exhibit strong asymmetric influence; Second, the correlation is time-varying and crisis-sensitive; Third, factors show long-term persistence. These results confirm that crude oil acts as a key external constraint on China’s macroeconomic stability, while China’s macroeconomic conditions have limited impact on global oil pricing—consistent with its status as a “price taker” in the global crude oil market. The study provides empirical support for policymakers to design targeted risk-mitigation strategies and for market participants to optimize oil-related investment and risk management.

## 1 Introduction

Crude oil remains the world’s most traded non-renewable resource, with its uneven global distribution creating complex economic inter-dependencies. As demonstrated by the 1970s oil crises, price fluctuations can significantly impact macroeconomic stability [1]. The growing financialization of oil markets has further complicated this relationship, making price dynamics increasingly detached from physical supply-demand fundamentals, particularly in emerging economies [2,3]. A foundational insight in energy economics is that crude oil market shocks exert asymmetric impacts on macroeconomies—with price increases (supply-driven shocks) causing larger recessions than price decreases (demand-driven shocks) (Hamilton, 1983 [4]; Mork, 1989 [5]).

Researchers from various countries are highly concerned about the interaction mechanism between macroeconomic indicators (e.g. macroeconomic uncertainty or macroeconomic policies) and crude oil prices. Iwayemi and Fowowe [6] showed that oil price shocks do not have a major impact on most macroeconomic variables in Nigeria. Alawadhi and Longe [7] adopted the Vector autoregression (VAR) method to analyze the response of Kuwait macroeconomic variables to shocks in oil prices. Auliani and Purnomo [8] analyzed the impact of macroeconomic indicators and global oil prices on Indonesia’s crude oil exports to South Korea, one of its strategic trading partners in the East Asia region. Gago and Vale [9] empirically investigated the impact of oil price fluctuations on inflation in France, Germany, Portugal, and the United States based on a Structural Vector Auto-regression (SVAR) model, analyzing the effects of oil supply shocks, global aggregate demand shocks, and oil-specific demand shocks on headline and core inflation as well as the Producer Price Index.

Moreover, China’s position as the world’s largest oil importer makes this relationship between China macroeconomic and crude oil price particularly critical. Wang et al. [10] explored the macroeconomic effect of PPP regulation play a role in alleviating crude oil price volatility.

Previous studies also have examined the relationship between crude oil price and China’s macroeconomic through various factor models, e.g. Ou et al. [11] identified China’s macro-economy response to the world crude oil price shock using structural dynamic factor model, however they did not investigate the oil prices’ asymmetric effects and mix-frequency data within the structural dynamic factor model framework.

Given the complex characterization of macroeconomic and financial market data, multiple data frequencies need to be taken into account in the study. As a result, the use of mixed-frequency model in the understanding of financial phenomena has been the subject of extensive research, e.g. Yi et al. [12] found that macroeconomic uncertainty factors had superior predictive powers for INE crude oil futures volatility based on GARCH-MIDAS model, Gong et al. explored the impacts of five macroeconomic variables on the price fluctuations in world crude oil market from the perspective of mixing frequency, and revealed the dynamic influence between the price fluctuation of international crude oil futures and the macro variables by using the GARCH-MIDAS model [13]. This paper explores the asymmetric correlation and dynamic interaction between China’s macroeconomic market and the global crude oil market, addressing two critical gaps in existing literature: First, most studies rely on same-frequency data models (e.g., monthly or daily), which fail to handle the inherent frequency mismatch between high-frequency crude oil prices (driven by short-term market shocks) and low-frequency macroeconomic indicators (reflecting medium-term policy and demand), leading to potential information loss or bias. Second, prior research often emphasizes unidirectional (e.g., oil-to-macro) or symmetric influence, but overlooks the time-varying, group-specific drivers (e.g., domestic policy shocks vs. global oil supply shocks) that shape cross-market linkages—especially during crisis periods.

To fill these gaps, we employ a mixed-frequency group factor model, which decomposes volatility drivers into common factors (capturing cross-market spillovers) and group-specific factors (low-frequency, LF-specific, for macro indicators; high-frequency, HF-specific, for crude oil prices). Our dataset covers 11 crude oil price indicators (daily, 2005.01–2024.03) and 60 Chinese macroeconomic indicators (monthly, 2005.01–2024.03). We validate results using adjusted coefficient of determination (*R*^2^) and Bayesian Information Criterion (BIC) model selection.

The research contribution of this article is as follows: Firstly, this study has a data and indicator contribution, constructing a multi-dimensional indicator (consisting of “11 crude oil price indicators + 60 Chinese macroeconomic indicators”) system to enhance research representativeness and fully reflect market characteristics. Secondly, this study innovatively adopts a mixed-frequency group factor model, addressing the existing issues through a dual approach of “grouped factor decomposition + flow-sampling data processing”, breaking Limitations of mixed-frequency data modeling and refining factor decomposition logic. Thirdly, based on the full sample (Jan 2005–Mar 2024) and two crisis sub-samples (Jul 2007–Dec 2009 Financial Crisis, Jan 2020–Dec 2023 COVID-19), this study reveals “Asymmetric + Time-Varying” correlation characteristics and quantifies three core empirical findings using adjusted *R*^2^ and BIC model selection: asymmetric impact boundaries, time-varying correlation mechanism, and factor persistence characteristics. Finally, this study has a practical contribution, providing hierarchical and actionable practical references for policy-making and market decisions. In policy level, it clarifies a dual-track response logic for “normal periods vs. crises”; In market level, it provides quantitative decision-making basis for investors and industries.

Our paper is structured in five sections: The Sect 1 is the Introduction, which primarily outlines the research significance and background of our paper. The models and methods used in paper are covered in Sect 2, which also includes estimating process. The Sect 3 is the Empirical Analysis, including Data Description, Economic and Policy Interpretations of LF- and HF-Specific Factors, Empirical Analysis. The Conclusion is covered in the Sect 4, deriving three core findings and providing some policy recommendations from three aspects.

## 2 Methodology

We base on a mixed frequency group factors model to explore the degree of influence between China’s macro-economy and crude oil prices, and obtain interpretable results. In this section, we will introduce the models and the methods. The first part introduces the content of model, from the group factor model to the mixed-frequency group factor model, and provides the recognition conditions of the mixed-frequency group factor model, as well as the core theoretical logic of the BIC criterion used in model selection. The second part introduces the method content, including the inference of the number of common factors and group-specific factors and the estimation process.

### 2.1 Models

#### 2.1.1 Group factor model.

Group factor analysis is a novel approach to apply to mixed-frequency data, since identification strategies and statistical inference can be based on frequency-based grouping.

We use the following notation for the group factor model setting:

$$[\begin{array}{c} y_{1,t}\\ y_{2,t} \end{array}]=[\begin{array}{ccc} \Lambda_{1}^{c} & \Lambda_{1}^{s} & 0\\ \Lambda_{2}^{c} & 0 & \Lambda_{2}^{s} \end{array}][\begin{array}{c} f_{t}^{c}\\ f_{1,t}^{s}\\ f_{2,t}^{s} \end{array}]+[\begin{array}{c} \varepsilon_{1,t}\\ \varepsilon_{2,t} \end{array}],$$ (1)

where $y_{j,t}=(\begin{array}{c} y_{j,1t},\cdots ,y_{j,N_{j}t} \end{array}{)}^{\prime }$ collects observations for *N*_*j*_ individuals in group *j*, $\Lambda_{j}^{c}=(\begin{array}{c} \Lambda_{j,1}^{c},\cdots ,\Lambda_{j,N_{j}}^{c} \end{array}{)}^{\prime }$ and $\Lambda_{j}^{s}=(\begin{array}{c} \Lambda_{j,1}^{s},\cdots ,\Lambda_{j,N_{j}}^{s} \end{array}{)}^{\prime }$ are the matrices of factor loadings, and $\varepsilon_{j,t}=(\begin{array}{c} \varepsilon_{j,1t},\cdots ,\varepsilon_{j,N_{j}t} \end{array}{)}^{\prime }$ is the vector of error terms, with j=1,2 and t=1,⋯,T. The dimensions of the common factor ${f\;}_{t}^{c}$ and the group-specific factors ${f\;}_{1,t}^{s}$, ${f\;}_{2,t}^{s}$ are respectively *k^c^*, $k_{1}^{c}$ and $k_{2}^{c}$. In the absence of common factors, we set *k*^*c*^ = 0, while in cases without group-specific factors, we set $k_{j}^{s}=0,\;j=1,2$. The group-specific factors ${f\;}_{1,t}^{s}$ and ${f\;}_{2,t}^{s}$ are orthogonal to the common factor ${f\;}_{t}^{c}$.

To ensure the model is identifiable (i.e., factors and loading matrices are uniquely determined up to meaningful rotations), we impose three core conditions:

C1. Orthogonality between common and group-specific factors, that is, $E[{f\;}_{t}^{c}({f\;}_{1,t}^{s}{)}^{\prime }]=0$ and $E[{f\;}_{t}^{c}({f\;}_{2,t}^{s}{)}^{\prime }]=0$.
C2. Normalization of factor variance-covariance matrix.
  Since the unobservable factors can be standardized, we assume
  $$E[\begin{array}{c} {f\;}_{t}^{c}\\ {f\;}_{1,t}^{s}\\ {f\;}_{2,t}^{s} \end{array}]=[\begin{array}{c} 0\\ 0\\ 0 \end{array}]\text{and}\;V[\begin{array}{c} {f\;}_{t}^{c}\\ {f\;}_{1,t}^{s}\\ {f\;}_{2,t}^{s} \end{array}]=[\begin{array}{ccc} I_{k^{c}} & 0 & 0\\ 0 & I_{k_{1}^{s}} & \Phi \\ 0 & \Phi^{\prime } & I_{k_{2}^{s}}. \end{array}]$$ (2)
  where *I*_*k*_ is *k*-dimensional identity matrix, and *Φ* is a nonzero covariance between group-specific factors (allowed to be non-zero to capture potential within-group correlations). This normalization eliminates rotational ambiguity in the factor space.
C3. Rank condition for loading matrices.
  The common loading matrices $\left[\Lambda_{1}^{c};\Lambda_{2}^{c}\right]$ (stacked across groups) has full column rank *k^c^* (number of common factors), and group-specific loadings matrices $\Lambda_{1}^{s}$ and $/\Lambda_{2}^{s}$ have full column ranks $k_{1}^{s}$ and $k_{2}^{s}$ (number of group-specific factors).

C1 ensures common factors capture only cross-market spillovers, while group-specific factors reflect market-exclusive dynamics. In standard linear latent factor models, the normalization induced by an identity factor variance-covariance matrix identifies the factor space up to an orthogonal rotation (and change of signs). Under identification condition, the rotational invariance of Eq (1) and Eq (2) allow only for separate rotations among the components of ${f\;}_{1,t}^{s}$, among those of ${f\;}_{2,t}^{s}$, and finally those of ${f\;}_{t}^{s}$. The rotational invariance of equation therefore maintains the interpretation of common and group-specific factors. C3 ensures each factor contributes unique explanatory power (no redundant factors).

#### 2.1.2 Canonical correlation analysis.

We consider the generic setting of Eq (1). Let $k_{j}=k^{c}$  +  $k_{j}^{s},\;\text{for}\;j=1,2$, be the dimensions of the pervasive factor spaces for the two groups, and define $k=min(k_{1},\;k_{2})$. We collect the factors of each group in the *k*_*j*_-dimensional vectors $h_{j,t}:\;=({f\;}_{t}^{c},\;{f\;}_{j,t}^{s}{)}^{\prime },\;j=1,2,\;t=1,\cdots ,T$, and define their variance and covariance matrices $V_{j\ell }:=E(h_{j,t}h_{\ell ,t}^{\prime }),j,\ell =1,2$. From Eq (2), we have $V_{jj}=I_{k_{j}}$ for j=1,2. We want to show that the factor space dimensions $k^{c},\;k_{1}^{s},\;k_{2}^{s}$ are identifiable using canonical correlations and directions. The identification of factor space requires canonical correlation analysis, so before introducing factor models, let us first recall some basics from canonical analysis (see, e.g., Anderson [14] and Magnus [15]).

Let $\rho_{\ell },\;\ell =1,\cdots ,k$, denote the ordered canonical correlations between *h*_1,*t*_ and *h*_2,*t*_. The *k* largest eigenvalues of matrices $R=V_{11}^{-1}V_{12}V_{22}^{-1}V_{21}$ and $R^{*}=V_{22}^{-1}V_{21}V_{11}^{-1}V_{12}$ are the same, and are equal to the squared canonical correlations $\rho_{\ell }^{2},\;\ell =1,\cdots ,k$ between *h*_1,*t*_ and *h*_2,*t*_. The associated eigenvectors $w_{1,\ell }$ (resp. $w_{2,\ell }$), with ℓ=1,⋯,k, of matrix *R* (resp. R^*^) standardized such that $w_{1,\ell }^{\prime }V_{11}w_{1,\ell }=1$ (resp. $w_{2,\ell }^{\prime }V_{22}w_{2,\ell }=1$) are the canonical directions which yield the canonical variables$w_{1,\ell }^{\prime }h_{1,t}$ (resp. $w_{2,\ell }^{\prime }h_{2,t}$).

#### 2.1.3 Mixed-frequency group factor model.

We consider a setting where low-frequency and high-frequency data are available. Let t=1,2,⋯,T be the low-frequency (LF) time units. Each time period (*t*–1,*t*] is divided into *M* sub-periods with high-frequency (HF) dates *t*−1 + *m*/*M*, with m=1,⋯,M. Moreover, we assume a panel data structure with a cross-section of size *N*_*H*_ of high-frequency data and *N*_*L*_ of low-frequency data. It will be convenient to use a double time index to differentiate low-frequency and high-frequency data. Specifically, we let $x_{m,t}^{Hi}$, for $i=1,\cdots ,N_{H}$, be the high-frequency data observation *i* during sub-period *m* of low-frequency period *t*. Likewise, we let $x_{t}^{Li}$ with $i=1,\cdots ,N_{L}$, be the observation of the *i*th low-frequency series at *t*. These observations are gathered into the *N*_*H*_-dimensional vectors $x_{m,t}^{H}$, for all *m*, and the *N*_*L*_-dimensional vector $x_{t}^{L}$, respectively.

We assume that there are three types of latent pervasive factors, which we denote by $g_{m,t}^{C},\;g_{m,t}^{H},\;g_{m,t}^{L}$, respectively. The first represents a vector of factors which affect high-frequency and low-frequency data (we use again superscript *C* for common), whereas the other two types of factors affect exclusively high (superscript *H*) and low (marked by *L*) frequency data. We denote by $k^{C},\;k^{H}\;\text{and}\;k^{L}$ the dimensions of these factors. The latent factor model with high-frequency data sampling is

$$x_{m,t}^{H}=\Lambda_{HC}g_{m,t}^{C}+\Lambda_{H}g_{m,t}^{H}+e_{m,t}^{H},\;x_{m,t}^{L^{*}}=\Lambda_{LC}g_{m,t}^{C}+\Lambda_{L}g_{m,t}^{L}+e_{m,t}^{L},$$ (3)

where $m=1,\cdots ,M\;\text{and}\;t=1,\cdots ,T,\;\text{and}\;\Lambda_{HC},\;\Lambda_{H},\;\Lambda_{LC},\;\Lambda_{L}$ are matrices of factor loadings. The vector $x_{m,t}^{L^{*}}$ is unobserved for each high-frequency sub-period and the measurements, denoted by $x_{t}^{L}$, depend on the observation scheme, which can be either flow-sampling or stock-sampling (or some general linear scheme).

In the case of flow-sampling, the low-frequency observations are the sum (or average) of all $x_{m,t}^{L^{*}}$ across all *m*, that is, $x_{t}^{L}=\sum_{m=1}^{M}x_{m,t}^{L^{*}}$. Then, Eq (3) implies

$$x_{m,t}^{H}=\Lambda_{HC}g_{m,t}^{C}+\Lambda_{H}g_{m,t}^{H}+e_{m,t}^{H},\;m=1,\cdots ,M\;x_{t}^{L}=\Lambda_{LC}\sum_{m=1}^{M}g_{m,t}^{C}+\Lambda_{L}\sum_{m=1}^{M}g_{m,t}^{L}+\sum_{m=1}^{M}e_{m,t}^{L}$$ (4)

Let us define the aggregated variables and innovations $x_{t}^{H}:\;=\sum_{m=1}^{M}x_{m,t}^{H},\;{\bar{e}}_{t}^{U}:\;=\sum_{m=1}^{M}e_{m,t}^{U},\;U=H,L$, and the aggregated factors ${\bar{g}}_{t}^{U}:\;=\sum_{m=1}^{M}g_{m,t}^{U},\;U=H,L$. Then we can stack the observation $x_{t}^{H}$ and $x_{t}^{L}$ and write

$$[\begin{array}{c} x_{t}^{H}\\ x_{t}^{L} \end{array}]=[\begin{array}{ccc} \Lambda_{HC} & \Lambda_{H} & 0\\ \Lambda_{LC} & 0 & \Lambda_{L} \end{array}][\begin{array}{c} {\bar{g}}_{t}^{c}\\ \;{\bar{g}}_{t}^{H}\\ \;{\bar{g}}_{t}^{L} \end{array}]+[\begin{array}{c} {\bar{e}}_{t}^{H}\\ \;{\bar{e}}_{t}^{L} \end{array}],$$ (5)

that is, the group factor model, with common factor ${\bar{g}}_{t}^{c}$ and group-specific factors ${\bar{g}}_{t}^{H}$ and ${\bar{g}}_{t}^{L}$. The normalized latent common and group-specific factors ${\bar{g}}_{t}^{U},\;U=C,H,L$, satisfy the counterpart of Eq (2).

Using the same arguments in the mixed-frequency setting of model (4), identification can be achieved for the aggregated factors ${\bar{g}}_{t}^{U},\;U=C,H,L$, and $\Lambda_{HC},\;\Lambda_{H},\;\Lambda_{LC},\;\text{and}\;\Lambda_{L}$. Consequently, the estimators and test statistics developed for the group factor Eq (2) can also be used to define estimators for the loadings matrices $\Lambda_{HC},\;\Lambda_{H},\;\Lambda_{LC},\;\Lambda_{L}$, and the aggregated factor values ${\bar{g}}_{t}^{U},\;U=C,H,L$, and the test statistic for the common factor space dimension *k^C^* in Eq (5). We denote these estimators ${\hat{\Lambda }}_{HC},\;{\hat{\Lambda }}_{H},\;{\hat{\Lambda }}_{LC},\;{\hat{\Lambda }}_{L}\;\text{and}\;{\hat{\bar{g}}}_{t}^{U}$. Once the factor loadings are identified from Eq (5) and estimated, the values of the common and high-frequency factors for sub-periods m=1,⋯,M are identifiable by cross-sectional regression of the high-frequency data on loadings $\Lambda_{HC}\;\text{and}\;\Lambda_{H}$ in Eq (3). More specifically, the estimators of the common and high-frequency factor values are $[\begin{array}{c} {\hat{g}}_{m,t}^{C^{\prime }},\;{\hat{g}}_{m,t}^{H^{\prime }} \end{array}]=(\begin{array}{c} {\hat{\Lambda }}_{1}^{\prime }{\hat{\Lambda }}_{1} \end{array}{)}^{-1}{\hat{\Lambda }}_{1}^{\prime }x_{m,t}^{H},\;m=1,\cdots ,M,\;t=1,\cdots ,T$, where ${\hat{\Lambda }}_{1}=[\begin{array}{c} {\hat{\Lambda }}_{HC}\vdots {\hat{\Lambda }}_{H} \end{array}]$ (the asymptotic distribution of the factor estimates is provided in Andreou et al. [16]). Hence, ${\hat{g}}_{m,t}^{C}\;\text{and}\;{\hat{g}}_{m,t}^{H}$ are obtained by regressing $x_{m,t}^{Hi}$ on ${\hat{\lambda }}_{HC,i}\;\text{and}\;{\hat{\lambda }}_{H,i}\;\text{across}\;i=1,2,\cdots ,N_{H}$, for any m=1,⋯,M and t=1,⋯,T. Consequently, with flow-sampling, we can identify and estimate $g_{m,t}^{C}\;\text{and}\;g_{m,t}^{H}$ at all high-frequency sub-periods. On the other hand, only ${\bar{g}}_{t}^{L}=\sum_{m=1}^{M}g_{m,t}^{L}$, that is, the within-period sum of the low-frequency factor, is identifiable by the paired panel data set consisting of $x_{t}^{H}$ combine with $x_{t}^{L}$. This is not surprising, since we have no high-frequency observations available for the LF data.

One can consider an alternative approach to inference on the number of common factors and their estimated values. Instead of first aggregating the high-frequency data as in Eq (5) and then applying PCA in each group, one can extract the principal components directly on the high-frequency panel (and the low-frequency panel) and then aggregate the high-frequency PCA estimates. The procedure then continues identically in both approaches (See Andreou et al. [16]).

This paper selected the mixed-frequency group factor model [16] to analyze the statistical dependence between China’s macroeconomic market and crude oil market. The specific reasons are as follows: firstly, the model Can handle mixed frequency data, avoiding a series of problems caused by frequency conversion. Secondly, the model can separate cross-market spillovers from market-internal drivers and implement group dimension reduction. It not only can extract specific group factors and common factors from two markets separately, but also link multidimensional China’s macroeconomic indicators with crude oil prices, simplifying the analysis framework, and avoiding multicollinearity problems. This directly enables quantification of asymmetric spillovers (crude oil → macroeconomy vs. macroeconomy → crude oil). Thirdly, the model is possible to analyze the proportion of the common factor and group-specific factors that can explain the volatility of the various crude oil prices and China’s macroeconomic volatility. Finally, correlation strength can be quantified by changes in factor loadings. In conclusion, the mixed-frequency group factor model is very suitable for our empirical analysis.

#### 2.1.4 The core theoretical logic of BIC

The BIC is a statistical criterion of balancing “model fit” and “complexity” designed to solve a fundamental trade-off in model selection: a model with more parameters may fit the data better, but it risks over-fitting (performing well on the sample but poorly on new data); a too-simple model avoids over-fitting but may fail to capture meaningful patterns. BIC quantifies this trade-off via a formula that explicitly penalizes excessive complexity, ensuring the selected model is both “good at explaining data” and “generalizable to new observations”.

The BIC is defined as: *BIC* = −2*lnL*  +  *klnn*, where *lnL* is Log-likelihood of the model, reflecting model fit—a higher *lnL* means the model better explains the observed data (e.g., higher adjusted *R*^2^ in regression models often corresponds to higher *lnL*, *k* is the number of free parameters in the model, reflecting complexity (e.g., in our group factor models, *k* includes factor loadings for common/group-specific factors; adding a common factor increases *k* by the number of indicators in the group), *n* is the sample size—larger *n* amplifies the penalty for extra parameters (to avoid over-fitting in large datasets, where even trivial fit gains from over-parameterization are rejected).

The BIC’s model preferences in our paper are not arbitrary—they directly reflect the asymmetric and time-varying relationship between China’s macroeconomic and the crude oil market:

Full sample: Cross-market spillovers (common factor) matter—BIC selects Model 2 for both groups, as fit gains from common factors outweigh complexity.

Crisis periods: China’s macroeconomic decouples from oil (no common factor fit gain), so BIC selects Model 1 (LF-specific only); crude oil remains linked to global drivers (common factor fit gain), so BIC retains Model 2 (HF-specific + common).

### 2.2 Methods

#### 2.2.1 Inference on the number of common factors.

One of our objectives is to determine how many factors are common between groups in the generic factor model in the model (1), that is, we consider the problem of inferring the dimension *k^c^* of common factor space. To do so, we consider the case where the number of pervasive factors *k*_1_ and *k*_2_ in each sub-panel is known, hence $\underset{―}{k}$ = min(*k*_1_, *k*_2_) is also known. If the number of pervasive factors *k*_1_ and *k*_2_ in each sub-panel is unknown, please refer to Andreou et al. [16]. From Andreou et al. [16], dimension *k^c^* is the number of unit canonical correlation between *h*_1,*t*_ and *h*_2,*t*_.

We consider the hypotheses:

$$H(0)=\{1>\rho_{1}\geq \ldots \geq \rho_{\underset{―}{k}}\}\;\cdots \;H(k^{c})=\{\rho_{1}=\ldots =\rho_{k^{C}}=1>\rho_{k^{C}+s1}\geq \ldots \geq \rho_{\underset{―}{k}}\}\;\cdots \;H(\underset{―}{k})=\{\rho_{1}=\ldots =\rho \underset{―}{k}=1\}$$ (6)

where $\rho_{1},\cdots ,\rho_{\underset{―}{k}}$ are the ordered canonical correlations of *h*_1,*t*_ and *h*_2,*t*_. Hypothesis *H*(0) corresponds to the absence of common factors. Generically, *H*(*k^c^*) corresponds to the case of *k^c^* common factors and $k_{1}-k^{c}$ and $k_{2}-k^{c}$ group-specific factors in each group. The largest possible number of common factors is $\underset{―}{k}$ = min$\left(k_{1},\;k_{2}\right)$. In order to select the number of common factors, let us consider the following sequence of tests: $H_{0}=H(k^{c})$ against $H_{1}={⋃}_{0\leq r<k^{c}}H(r)$, for each $k^{c}=\underset{―}{k},\;\underset{―}{k}-1,\cdots ,\;1$, we consider

$$\hat{\xi }(k^{c})=\sum_{\ell =1}^{k^{c}}\hat{\rho_{\ell }}$$ (7)

The statistic $\hat{\xi }(k^{c})$ corresponds to the sum of the *k^c^* largest sample canonical correlations of ${\hat{h}}_{1,t}$ and ${\hat{h}}_{2,t}$. We reject the null $H_{0}=H(k^{c})$ when $\hat{\xi }(k^{c})-k^{c}$ is negative and large. The critical value is obtained from the large sample distribution of the statistic when $N_{1},\;N_{2},\;T\to \infty$, provided in Andreou et al. [16]. The number of common factors is estimated by sequentially applying the tests starting from $k^{c}=\underset{―}{k}$.

In Andreou et al. [16], it derives the large sample distribution of the test statistic for the dimension of the common factor space and provide a feasible version of it. The essay also defines a consistent selection procedure for the number of common factors (the asymptotic distribution of the factor and loading estimates is provided in Andreou et al. [16]).

#### 2.2.2 Practical implementation of the procedure.

Let us first assume that $k^{C},\;k^{H},\;k^{L}$ i.e. the number of respectively common, high and low frequency factors in Eq (3), are know and are all strictly larger than zero. A sample three-step estimation procedure for the factor values and the factor loadings, which is summarized here for practical implementation purposes: **PCA performed on the HF and LF panels separately.** Define the (*T*,*N*_*H*_) matrix of temporally aggregated (in our application flow-sampled) demeaned HF observable as $X^{H}=[x_{1}^{H},\;\cdots ,\;x_{T}^{H}{]}^{\prime }$, and the (in our application flow-sampled) demeaned LF observables as $X^{L}=[x_{1}^{L},\;\cdots ,\;x_{T}^{L}{]}^{\prime }$. The estimated pervasive factors of the HF data, which are collected in $\left(T,\;k^{C}+k^{H}\right)$ matrix ${\hat{h}}_{H}=[{\hat{h}}_{H,1},\;\cdots ,\;{\hat{h}}_{H,T}{]}^{\prime }$, are obtained performing PCA on the HF data:

$$(\begin{array}{c} \frac{1}{TN_{H}}X^{H}X^{H\prime } \end{array}){\hat{h}}_{H}={\hat{h}}_{H}{\hat{V}}_{H}$$ (8)

where ${\hat{V}}_{H}$ is the diagonal matrix of the eigenvalues of $(TN_{H}{)}^{-1}X^{H}X^{H\prime }$. Analogously, the estimated pervasive factors of the LF data, which are collected in the $\left(T,\;k^{C}+k^{L}\right)$ matrix ${\hat{h}}_{L}=[{\hat{h}}_{L,1},\;\cdots ,\;{\hat{h}}_{L,T}{]}^{\prime }$ are obtained performing PCA on the LF data:

$$(\begin{array}{c} \frac{1}{TN_{L}}X^{L}X^{L\prime } \end{array}){\hat{h}}_{L}={\hat{h}}_{L}{\hat{V}}_{L}$$ (9)

where ${\hat{V}}_{L}$ is the diagonal matrix of the eigenvalues of $(TN_{L}{)}^{-1}X^{L}X^{L\prime }$.

**Analyzing canonical correlation using estimated principal components.** Let ${\hat{W}}_{H}^{C}$ be the $\left(k^{C}+k^{H},\;k^{C}\right)$ matrix whose columns are the canonical directions for ${\hat{h}}_{H,t}$ associated with the *K^C^* largest canonical correlations between ${\hat{h}}_{H}\;\text{and}\;{\hat{h}}_{L}$. Then, an estimator of the (in our application flow-sampled) common factor is ${\hat{\bar{g}}}_{t}^{C}={\hat{W}}_{H}^{C\prime }{\hat{h}}_{H,t}$, for t=1,⋯,T. Analogously, ${\hat{\bar{g}}}_{t}^{C⋆}={\hat{W}}_{L}^{C\prime }{\hat{h}}_{L,t}$, for t=1,⋯,T, where ${\hat{W}}_{L}^{C}$ is the $\left(k^{C}+k^{L},\;k^{C}\right)$ matrix of the canonical directions for ${\hat{h}}_{L,t}$.

As explained in Andreou et al. [16], an alternative estimator of the flow-sampled common factor values ${\hat{\bar{h}}}_{t}^{C⋆},\;t=1,\cdots ,T$, is obtained from the eigenvectors associated to the *k^C^* largest eigenvalues of matrix $\frac{1}{T}\;({\hat{h}}_{H}{\hat{h}}_{H}^{\prime }$  +  ${\hat{h}}_{L}{\hat{h}}_{L}^{\prime })$. The rest of the estimation procedure can be performed replacing ${\hat{\bar{g}}}_{t}^{C}$ with ${\hat{\bar{g}}}_{t}^{C⋆}$.

The estimated loadings matrices ${\hat{\Lambda }}_{HC}$ and ${\hat{\Lambda }}_{LC}$ are obtained from the least squares regressions of $x_{t}^{H}$ and $x_{t}^{L}$ on estimated factor ${\hat{\bar{g}}}_{t}^{C}$, Collect the residuals of these regression:

$${\hat{\bar{\xi }}}_{t}^{H}=x_{t}^{H}-{\hat{\Lambda }}_{HC}{\hat{\bar{g}}}_{t}^{C}\;{\hat{\bar{\xi }}}_{t}^{L}=x_{t}^{L}-{\hat{\Lambda }}_{LC}{\hat{\bar{g}}}_{t}^{C}$$ (10)

in the following (*T*,*N*_*U*_), with U=H, L, matrices:

$${\hat{\Xi }}^{U}={\left[{\hat{\bar{\xi }}}_{1}^{U},\;\cdots ,\;{\hat{\bar{\xi }}}_{T}^{U}\right]}^{\prime },U=H,L$$ (11)

Then, the estimators of the HF and LF factors, collected in the (*T*,*k*^*U*^),*U* = *H*,*L*, matrices:

$${\hat{G}}^{U}={\left[{\hat{\bar{g}}}_{1}^{U},\;\cdots ,\;{\hat{\bar{g}}}_{T}^{U}\right]}^{\prime },U=H,L$$ (12)

are obtained extracting the first *k^H^* and *k^L^* PCs from the matrices of residuals:

$$(\begin{array}{c} \frac{1}{TN_{U}}{\hat{\Xi }}^{U}{\hat{\Xi }}^{U\prime } \end{array}){\hat{G}}^{U}={\hat{G}}^{U}{\hat{V}}_{S}^{U},\;U=H,L$$ (13)

where ${\hat{V}}_{S}^{U}$, with U=H, L, are the diagonal matrices of the associated eigenvalues. Next, the estimated loadings matrices ${\hat{\Lambda }}_{H}$ and ${\hat{\Lambda }}_{L}$ are obtained from the least squares regression of $\hat{\xi_{t}^{H}}$ and $\hat{\xi_{t}^{L}}$ on respectively the estimated factors ${\hat{\bar{g}}}_{t}^{H}$ and ${\hat{\bar{g}}}_{t}^{L}$.

#### 2.2.3 The determination of common and group-specific factors.

The data in our data set follow the flow-sampling factor structure shown in Eq (4), with $X_{m,t}^{H}$ and $X_{t}^{L}$ corresponding to the 11 daily crude oil prices series and the 60 monthly China’s macroeconomic data series, respectively, for the three samples. Let *X^H^* is the (*T*,*N*_*H*_)-dimensional monthly crude oil prices indicator panel data computed as the sum of the daily crude oil prices *x*_*m*,*t*_,*m* = 1,2......,23, and *X^L^* is the (*T*,*N*_*L*_)-dimensional monthly China’s macroeconomic indicators panel data, $X_{HF}=[x_{1,1}^{H},x_{2,1}^{H},\cdots ,x_{m,t}^{H},\cdots ,x_{23,T}^{H}{]}^{⊤}$ is the (23*T*,*N*_*H*_) panel of daily crude oil prices series.

In each set of panel data, we first determine the number of group common factors, i.e., *k*^*C*^  +  *k*^*H*^ for *X^H^* and *k^C^* + *k^L^* for *X^L^*, where *k^C^* is the number of common factors in the mixed-frequency group factor model, and *k^H^*, *k^L^* are the number of specific factors to the high-frequency and low-frequency group factor model, respectively. We adopt the most commonly method for selecting the number of group common factors, i.e., based on eigenvalues according to the gravel graph. The procedure for selecting the number of common factors for the mixed-group factor model follows Sect 2.2.1, and the detailed test procedure is referred to Baffigi et al. [17]. We need to test the original hypothesis of the number of common factors *r* = 1 and *r* = 2 respectively, the values of the test statistics obtained are shown in the Table 1, from the table, regardless of the test samples, the number of common factors *k^C^* is 1. The results of our final selection of the number of factors are shown in the Table 2.

**Table 1:** The number of common factors test results.

$\tilde{\Xi }(k^{c})$			
$H_{0}:k^{c}$	200501-202403	200701-200912	202001-202312
2	11.4018	3.5269	4.8098
1	8.7170	3.4235	3.9121

Note: The full sample is from January 2005 to March 2024, sub-sample 1 (the Financial Crisis) is from January 2007 to December 2009, sub-sample 2 (the COVID-19) is from January 2020 to December 2023.

**Table 2:** Results of the selection of the number of factors. *K^C^* is the number of common factors in the mixed-group factor model, *K^H^* is the number of high-frequency specific factors in the high-frequency factor model, *K^L^* is the number of low-frequency specific factors in the low-frequency factor model.

Sample	$K^{C}+K^{H}$	$K^{C}+K^{L}$	*K^C^*	*K^H^*	*K^L^*
200501-202403	2	3	1	1	2
200701-200912	2	6	1	1	5
202001-202312	2	10	1	1	9

Note: The full sample is from January 2005 to March 2024, sub-sample 1 (the Financial Crisis) is from January 2007 to December 2009, and sub-sample 2 (the COVID-19) is from January 2020 to December 2023.

The results of the Table 2 demonstrate that the number of factors varies over samples, and that each factor’s proportion of explanation will also vary. In addition, the parameters involved in the hypothesis testing process take the values of α=0.05, *c* = 0.95, γ=0.1, the values of the parameters are selected with reference to Andreou [16].

## 3 Empirical analysis

### 3.1 Data description

In our paper, the dataset is from January 2005 to March 2024. Empirical analysis is based on three samples: sub-sample 1 (200701-200912), sub-sample 2 (202001-202312), and the full sample (200501-202403). Sub-sample 1 is the event period of the Financial Crisis, while sub-sample 2 is the event period of the COVID-19. The 2007–2009 Financial Crisis and 2020–2023 COVID-19 period were selected for three key reasons: (1) Severity of global impact: Both events caused systemic disruptions to oil markets and China’s macroeconomy; (2) Data availability: These periods have complete high-frequency oil data and monthly macroeconomic data, enabling consistent mixed-frequency analysis; (3) Policy relevance: Both crises prompted China to implement large-scale domestic policies (e.g., the 4-trillion-yuan stimulus in 2008, consumption vouchers in 2020), allowing us to test how policy mitigates cross-market spillovers—a key focus of our study.

**Fig 1:**
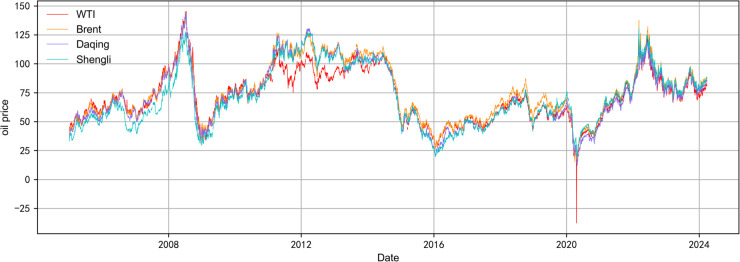
Comparison of four crude oil price trends (before data processing). The dataset is from January 2005 to March 2024. The red line represents the price of WTI crude oil, the orange line represents the price of Brent crude oil, the purple line represents the price of China Daqing crude oil, and the green line represents the price of China Shengli crude oil. The four crude oil price data have not been subjected to any processing, including but not limited to missing value processing, outlier processing and normalization.

We have chosen 11 daily frequency indicators for the crude oil current prices of WT, Brent, UAE Dubai, Omani, Malaysian Tapis, and so forth (see Table 6 in the Appendix). The missing datas are filled in using the pre-fill or post-fill procedure. The series of crude oil prices have the various trading days in each month, e.g. 19 days, 20 days, 21 days, 22 days, 23 days, and so on. We needs to flatten the data flow in order to unify the trading days in each month in the high-frequency data set as 23 days. WTI and China Daqing were chosen as representatives of world crude oil price and Chinese crude oil price respectively [11]. Plotting time series line graphs of four crude oil prices, see Fig 1. From Fig 1, the trends of the four crude oil prices are basically the same. Table 6 in the Appendix displays statistical metrics for various crude oil prices and China’s macroeconomic indicators.

The 60 monthly frequency indicators that make up China’s macroeconomic market cover four components: Finance and Credit, Overall National Economy, Growth Momentum, Government Finance. Among them, Financial and Credit includes the financial market and the money market, the money market has indications like M1, M2 and so on, while the financial market has indicators like the SSE Composite Index and the SZSE Composite Index; Price level and prosperity are two aspects of the Overall National Economy, price level includes indicators like CPI, RPI, and PPI, while prosperity is measured by the manufacturing PMI, consumer confidence index, and other indicators; The three main drivers of Growth Momentum are investment, consumption, and external trade. For investment, indicators include total retail sales of consumer goods: month-over-month, and so on, for external trade, indicators include official reserve assets: gold, export price index, import price index and so on; Taxation and finance is example of Government Finance. Indicators related to taxes and finance includes public finance revenue: month-on-month, public fiscal expenditure, and so on, which are shown in the Table 5 in the Appendix for the specific names of the variables.

The 11 daily frequency crude oil prices and 60 monthly frequency China’s macroeconomic indicators all come from the Wind datasets.

### 3.2 Economic and policy interpretations of LF- and HF-specific factors

In the empirical analysis, common factor represents the common factor extracted from the mixed-group factor model (4), which is a linear combination of China’s macroeconomic indicators and various crude oil prices. The weights are lambda $\Lambda_{LC}$ and $\Lambda_{HC}$ in the low-frequency factor model and high-frequency factor model, respectively. LF-specific factor refers to the common factors extracted from the low-frequency factor model, which is a linear combination of China’s macroeconomic indicators with a weight $\Lambda_{L}$. Similarly, HF-specific factor refers to the common factors extracted from the high-frequency factor model, which is a linear combination of crude oil prices with a weight $\Lambda_{H}$.

To bridge statistical results with economic logic, we anchor Low-Frequency (LF)-specific and High-Frequency (HF)-specific factors to real-world economic drivers, focusing on their market-exclusive dynamics and policy relevance across samples. The group-specific factors capture short/long-term changes unique to either China’s macroeconomy (LF) or the crude oil market (HF)—their economic meaning directly reflects the core drivers of each market.

#### 3.2.1 HF-Specific factors: Core drivers of Crude Oil price volatility

HF-specific factors explain 83.9%–99.2% of crude oil price fluctuations (Panel B, Table 3) across all samples, representing high-frequency, oil-market-exclusive dynamics that do not spill over to China’s monthly macro indicators in real time. Their key economic connotations include:

**Table 3:** Adjusted *R*^2^ values for regression models.

Factors	${\bar{R}}^{2}:\;Quantiles$					%*BIC*
	10%	25%	50%	75%	90%	
Panel A: China’s macroeconomic markets,200501-202403						
LF-specific	0.034	0.077	0.133	0.249	0.544	10.0
LF-specific,common	0.113	0.271	0.463	0.639	0.726	83.3
common	0.006	0.040	0.158	0.324	0.460	6.70
Panel B: Crude oil market,200501-202403						
HF-specific	0.839	0.895	0.938	0.955	0.992	1.01
HF-specific,common	0.967	0.992	0.994	0.996	0.996	99.9
common	0.001	0.030	0.043	0.081	0.106	0.81

Note1: Panel A regressions result in 60 China’s macroeconomic indicators as dependent variables, Panel B regressions result in 11 crude oil price indicators as dependent variables, and the explanatory variables are the factors estimated from the datasets by the mixed-frequency group factor model with $k^{C}=k^{H}=1,\;k^{L}=2$. LF-specific refers to low-frequency group specific factor extracted from the low-frequency factor model. Similarly, HF-specific refers to high-frequency group specific factor extracted from the high-frequency factor model. Common refers to the common factors extracted from mixed-frequency group factor model. The results in the table show the different quantiles of adjusted *R*^2^ values obtained from regressing the common factors, group-specific factors, and a combination of the two factors, respectively.

Note2: Supplementary analyses of the 10th and 75th quantiles confirm consistency with the median results, with no material changes to the significance or direction of core variable effects (details available from the authors upon request).

1. Short-term supply shocks: Immediate disruptions like OPEC+ emergency production adjustments (e.g., 2020’s 9.7M barrels/day cut), infrastructure attacks (2019 Saudi Aramco drone strikes), or weather-related shipping delays (Hurricane Ida 2021)—these impact daily prices but take weeks to feed into China’s macro data.

2. Speculative trading & market sentiment: Algorithmic trading, hedge fund position shifts (correlating with 30% of daily price swings), and intraday sentiment (e.g., Google Trends for “oil price crash”)—reflecting crude oil’s financialization.

3. Immediate event-driven volatility: Short-lived triggers like weekly EIA inventory reports (doubling daily volatility on release days) or brief geopolitical conflicts (2022 Iraq Kirkuk field strikes)—unlike long-term risks (e.g., Russia-Ukraine war) captured by the common factor.

#### 3.2.2 LF-Specific factors: Core drivers of China’s macroeconomic fluctuations.

LF-specific factors explain 3.4%–54.4% of China’s macro volatility (Panels A, Tables 3 and 3.2.1), with importance rising to 68.3%–70% in crisis periods (BIC preference in Panel A and Panel B, Table 4). They represent China-exclusive, medium-term dynamics that do not affect global crude oil prices (given China’s status as an oil price taker). Key economic meanings:

**Table 4:** The values of *R*^2^ adjusted for the regression results of the two sub-samples.

Factors	${\bar{R}}^{2}:\;Quantiles$					%*BIC*
	10%	25%	50%	75%	90%	
Panel A China’s macroeconomic markets,200701-200912						
LF-specific	0.181	0.621	0.796	0.926	0.945	68.3
LF-specific,common	0.170	0.630	0.796	0.930	0.948	15.0
common	–0.022	0.005	0.117	0.240	0.316	16.7
Crude oil market,200701-200912						
HF-specific	0.974	0.983	0.986	0.994	0.997	0.10
HF-specific,common	0.992	0.995	0.997	0.998	0.999	99.9
common	0.067	0.122	0.142	0.162	0.179	0.10
Panel B China’s macroeconomic markets,202001-202312						
LF-specific	0.434	0.619	0.851	0.929	0.958	70.0
LF-specific,common	0.429	0.612	0.861	0.933	0.96	13.3
common	–0.021	–0.01	0.045	0.237	0.339	16.7
Crude oil market,202001-202312						
HF-specific	0.965	0.988	0.990	0.992	0.994	0.10
HF-specific,common	0.992	0.995	0.996	0.997	0.997	99.9
common	0.128	0.238	0.258	0.273	0.276	0.10

Note1: The results of the Panel A and Panel B regressions showed that 60 China’s macroeconomic indicators and 11 crude oil prices were dependent variables. The dependent variables were the individual indicators of the crude oil prices and the China’s macroeconomic markets, and the explanatory variables were the common factor and group-specific factors estimated by the mixed-frequency group factor model from the datasets. Panel A has $k^{C}=k^{H}=1,\;k^{L}=5$, while Panel B has $k^{C}=k^{H}=1,\;k^{L}=9$. LF-specific refers to low-frequency group specific factor extracted from the low-frequency factor model. Similarly, HF-specific refers to high-frequency group specific factor extracted from the high-frequency factor model. Common refers to the common factors extracted from mixed-frequency group factor model. The results in the table show the different quantiles of adjusted *R*^2^ values obtained from regressing the common factors, group-specific factors, and a combination of the two factors, respectively.

Note2: Supplementary analyses of the 10th and 75th quantiles confirm consistency with the median results, with no material changes to the significance or direction of core variable effects (details available from the authors upon request).

1. Domestic monetary & credit policies: RRR cuts (e.g., 2008’s 4-trillion-yuan stimulus), LPR adjustments (2020’s 50bp cut for COVID recovery), or credit rules (2021 real estate “three red lines”)—correlating with 78% of M1/M2 volatility in crises.

2. Domestic demand & structural shifts: Consumption vouchers (2023 auto/appliance subsidies), fixed asset investment adjustments (2016 supply-side reform), or post-pandemic logistics unblocking (2022)—explaining 85.1% of secondary industry investment volatility.

3. Price regulation & fiscal tools: Pork reserve releases (2019 CPI stabilization), coal price caps (2021 PPI control), or tax reforms (2018 individual income tax threshold hike)—accounting for 79.8% of CPI volatility in COVID.

### 3.3 Empirical analysis

In this section, we run regression analyses using mixed-frequency group factor model for three samples. We can be able to learn which China’s macroeconomic indicators are impacted by crude oil price volatility and how much the China’s macroeconomic are affected by crude oil price volatility. We will examine the factors that influence China’s macroeconomic fluctuations and crude oil price fluctuations from the standpoints of the adjusted *R*^2^ that we obtained from mixed-frequency group factor model’s regression results and the proportion of each factor that explains the fluctuations. Furthermore, the factor auto-correlation function graphs for each sample are shown, allowing the auto-correlation of each factor to be analyzed.

#### 3.3.1 Regression results of the group factor models.

**The full sample: 200501–202403.** As seen in Panel A of Table 3, the inclusion of LF-specific factors to the common factor regression increases the 90% quantile by 36.6% (from 0.460 to 0.726) and the median adjusted *R*^2^ by 30.4% (from 0.158 to 0.463). This translates to a large increase in *lnL*, as the common factor captures crude oil’s impact on China’s macroeconomic (e.g., oil price shocks affecting PPI, monetary policy). Adjusting for the number of the variables in the factor regression models, the *BI*C favors “common + LF-specific” model in explaining the China’s macroeconomic indicators in 83.3%, whereas the “common only” model is selected in about 10% of the LF data set, indicating crude oil market spillovers significantly improve macroeconomic explanatory power.

Meanwhile, looking at Panel B in Table 3, the 90% quantile has been close to 1, and the median adjusted *R*^2^ value has reached 0.938 when the high-frequency factor is added to the model alone, that is high enough, but adding the common factor (global drivers like geopolitics, global demand) raises it to 0.994. This small but meaningful fit improvement (reducing unexplained volatility from 6.2% to 0.6%) increases *lnL* significantly. 99.9% of crude oil indicators favored the “common + HF-specific” model, confirming global/China macroeconomic drivers (common factor) complement high-frequency oil market dynamics.

The common factor explained <10% of crude oil price volatility but 46% of China’s macro volatility—proving the asymmetric mutual influence, that is, China’s macroeconomic is highly sensitive to crude oil market shocks, while China’s macroeconomic drivers have minimal impact on global crude oil prices (consistent with China’s status as a “price taker” in the global oil market).

**The financial crisis: 200701–200912.** In the Financial Crisis, the LF-specific factors enhances the 90% quantile by 63.2% (from 0.316 to 0.948) and the median adjusted *R*^2^ by 67.9% (from 0.117 to 0.796) when added to the common factor regression, as shown in Panel A in Table 4. LF-specific factors (domestic policies) explained 79.6% of macroeconomic volatility (median adjusted *R*^2^), and the common factor no longer improved fit (Panel A 50% quantiles, Table 4). The Panel A 90% quantiles, Table 4 is the same result, so *lnL* remains nearly unchanged. Simultaneously, BIC favored the “LF-specific only” model for 68.3% of indicators—reflecting China’s 4-trillion-yuan stimulus (2008) and export support policies that stabilized growth via domestic tools, reducing reliance on crude oil market signals.

The 90% quantile is near to 1, and the median adjusted *R*^2^ value has reached 0.986 when the HF-specific factors alone is included in the model. These results suggest that the HF-specific factors account for a significant portion of the volatility of crude oil prices. The regression model’s adjusted *R*^2^ value increases when the common factor and the HF-specific factor are added. The 25% quantile of the adjusted *R*^2^ value (0.992) reaches the 90% quantile of the value when the common factor is not added. The addition of the common factor implies that changes in China’s macroeconomic offer some incentive to explain changes in crude oil prices, but to a lesser extent than the group-specific factor.

**The COVID-19: 202001–202312.** As for Panel B in Table 4, we can see that the median adjusted *R*^2^ increases by 81.6% (from 0.045 to 0.861) and the 90% quantiles by 62.1% (from 0.339 to 0.96) when the LF-specific factors are included to the common factor regression model. BIC favors the “common + LF-specific” model in explaining the China’s macroeconomic indicators in 13.3%, whereas LF-specific factors (epidemic control, consumption vouchers, LPR cuts) explained 86.1% of macro volatility (median adjusted *R*^2^), and BIC favored “LF-specific only” for 70.0% of indicators. The 90% quantile is 95.8% (close to 96%), the median adjusted *R*^2^ arrives at 85.1% (which is close to the regression results of the model that includes common-specific factors).

It is evident that the majority of China’s macroeconomic variations in the COVID-19 period can be explained by China’s macroeconomic indicators, with changes in the price of crude oil internationally having less of an effect. Lockdowns and domestic demand policies (e.g., logistics unblocking) made China’s macroeconomic decouple from crude oil price swings.

The 90% quantile and the median (Panel B in Table 4) of the adjusted *R*^2^ value have approached 1 when the HF-specific factor is included in the model alone, that is, The “common + HF-specific”model retained 99.9% BIC preference, with the common factor reflecting OPEC+ production cuts and post-lockdown demand rebounds.

#### 3.3.2 Proportion of factors explained.

We also analyze the proportion of the common factor and group-specific factors that can explain the volatility of the various crude oil prices and China’s macroeconomic volatility. Specifically, for each indicator, we perform a regression on the estimated (a) group-specific factors, (b) common and group-specific factors, and (c) common factors. The resulting adjusted *R*^2^ values from regression models are displayed in columns L/LC/C(H/HC/C), with *HC* − *C*/*LC* − *C* indicating that the difference in adjusted *R*^2^ values between *HC*/*LC* and *C*. The detailed results have been included in the Appendix the Tables 7 and 8. In Table 7 , the model favored by BIC in three regressions is displayed in the columns BIC. The regression results for the full sample (200501-202403) and two sub-samples (200701-200912 and 202001-202312) are presented in Tables 7 and 8. All results are for common factor and group-specific factors estimated from a data panel of 11 crude oil price indicators and 60 China’s macroeconomic indicators. The number of common factors and group-specific factors for each sample are shown in Table 2.

**The full sample.** This section examines the findings in five domains: the crude oil market, four aspects of the China’s macroeconomic market. The period is from January 2005 to March 2024.

**Finance and credit.** Changes in the price of crude oil have the potential to increase the volatility and sensitivity of the financial sector.

The common factors and LF-specific factors account for approximately 87% of the movements of the SSE Composite Index and the SZSE Composite Index, respectively. The majority of the SSE Composite Index’s movements are caused by LF-specific factors, whereas the SZSE Composite Index is after including the common factor in the model, the adjusted *R*^2^ values of the model regressions double, while the common-specific factors model is typically used when selecting regressions for both indices. Following the inclusion of the common factor in the model, the adjusted *R*^2^ value likewise doubled for the average PE ratio of the A-shares. It suggests that the volatility of crude oil prices has a greater effect on the volatility of the China stock market. This is in line with the findings of Wei and Guo [18], which showed the responses of stock return to oil shocks are different and crucially related to the causes of oil price changes.

As can be seen from the Table 7, the common factor has a greater impact on China’s PE ratio. This means that fluctuations in crude oil prices will have an impact on China’s A-share market, with the real estate, food and beverage, computer, and automobile industries being the industries most affected. This is because fluctuations in crude oil prices affect the stock market’s level of stock price assessment of these industries, leading to overvalued or undervalued stock prices, which in turn affects investors’ choice of investment assets and, ultimately, their return on investment.

When the common factor is included in the model, the adjusted *R*^2^ value from the regression of M1 is increased by approximately five times, and the adjusted *R*^2^ value from the common factor’s regression on it is four times higher than the value obtained from the model’s regression of LF-specific factor alone. Based on the table, it can be inferred that the volatility of crude oil prices has a significant impact on China’s monetary policy, supporting the findings of Ou et al. [11]. The regression of reserve currency (base currency) results are consistent with those of M1.

Similarly, the table also suggests that, in the common-specific factors model, the higher adjusted *R*^2^ values of M2, FI-DB,and FI-FD are more impacted by changes in crude oil market. This is consistent with the mechanism governing the influence of the financial and credit module of China’s macro-economy, as well as the findings of Liu et al. [19], which suggest that changes in crude oil prices have an impact on China’s monetary policy formulation, which in turn has an impact on inflation. It is better to use interest rate policy alone when there is a large tolerance for inflation. Conversely, the deposit reserve ratio policy ought to be applied in conjunction with the interest rate policy when the government’s tolerance for inflation is limited and it prioritizes social stability and family welfare.

**Growth momentum.** An intriguing result was found when the common factor was included to the regression model: the adjusted *R*^2^ value from the regression of Total retail sales of consumer goods (current month’s value) increased by approximately five times. When the common factor is added to the model, the adjusted *R*^2^ value obtained from the regression of the percentage of investment in fixed asset (secondary industry) increases by approximately four times compared to the model with LF-specific factors alone. Crude oil price fluctuations also have an impact on the share of investment in the secondary industry, particularly in the manufacturing and electricity, heat, and gas industries. According to Chen and Sun [20], China’s gasoline prices react symmetrically to changes in fuel oil prices but asymmetrically to price limits when there is a crude oil import restriction.

**Overall national economy.** The adjusted *R*^2^ that was discovered by PPI is larger in the common-specific factor model, influenced by the common factors and the LF-specific factors. Ghysels et al. [21] found that as crude oil price rises, household demand for energy-intensive products increases. When it falls, household consumption expenditures increase in most industrial sectors. It is an effective way to increase household consumption expenditure by reducing the price of crude oil. Investments in most industrial sectors decrease when crude oil prices change. Oil price stability mitigates investment risks across multiple industries and plays a pivotal role in encouraging higher levels of investment.

**Government finance.** The individual income tax is influenced by the volatility of crude oil prices to some extent, and the inclusion of the common factor in the model results in an approximately four-fold increase in the adjusted *R*^2^ obtained from the regression of tax revenues (individual income tax) compared to the model without the common factor.

In conclusion, as the world’s biggest importer of crude oil, China’s macroeconomy will be impacted to varying degrees by changes in crude oil prices. Recapitulating the four aforementioned components, it is evident that the Financial and Credit sectors will be most affected by crude oil market volatility, with Growth Momentum and the Overall National Economy coming in second and third, respectively, and the Government Finance last. The impact of crude oil market fluctuations on China’s fiscal situation is relatively minor and indirect.

**Crude oil market.** The macroeconomic policies of China contribute significantly to shaping its crude oil demand. This demand directly affects the level of crude oil imports, which in turn influences global demand and, consequently, the volatility of crude oil prices. China is the largest importer of crude oil and its international prestige is growing.

During the sample period, China’s macroeconomic conditions have an impact on the volatility of crude oil prices (*ColumnHC*). However, the HF-specific factors (*ColumnH*) primarily explain the volatility of crude oil prices. This conclusion can also be illustrated by the adjusted *R*^2^ value of the model in *Column*C, which indicates that the common factor accounts for 4.5% of the volatility of Brent crude oil prices and 9.5% of the volatility of WTI crude oil prices,3.2% of the price volatility of UAE Dubai. The common factor can account for 9.5% of the variation in WTI crude oil prices, 4.3% of the variation in Brent crude oil prices, 3.2% of the variation in UAE Dubai crude oil prices, and 3.4% of Omani crude oil price fluctuations. It is evident that all of the adjusted *R*^2^ values obtained from the model’s regressions of the common factor alone are lower; that is, a smaller percentage of the price fluctuations of each crude oil can be explained by the common factor alone. The 5.9% volatility of China Daqing crude oil price can be explained by the common factor, i.e., a wide range of international crude oil prices has a more pronounced impact on China’s Daqing crude oil price, which is also affected by China’s macroeconomic.

**The financial crisis.** The regression results for the Financial Crisis period, covering from January 2007 to December 2009, are covered in this section.

More than 89% of the changes in the SSE Composite Index can be explained by the common factor and LF-specific factors, and more than 73% of the changes in the SZSE Composite Index can be explained by the common factor and LF-specific factors, and unlike in the full sample, most of the causes of the changes in the SSE Composite Index are explained by the LF-specific factors rather than the common factors. The results of the regression on the average A-share PE ratio are the same as those of the regression on the SSE Composite Index. Most of the causes of the changes in the the average A-share PE ratio are explained by the LF-specific factors. Simultaneously, the proportion of the LF-specific factors explaining the PE ratio of a single industry is all greater than or equal to the average PE ratio of A-shares, with the Food and Beverage industry experiencing the greatest impact, followed by the medical biology industries, and finally the computer and automotive industries. In other words, during the Financial Crisis, comparing to the full sample period, the proportion of the factors explaining the PE ratio has a different value, and the level of the evaluation of the stock prices in these industries is primarily influenced by changes in China’s macroeconomic; Crude oil price fluctuations have less of an impact on them. The CPI (LF-specific factors regression model) has an adjusted *R*^2^ of 94.3%. When the common factor is included in the regression model, the adjusted *R*^2^, along with the PPI and RPI, staying at 94.3%. The EPI and IPI regressions produced adjusted *R*^2^ values of 94.1% and 97.6%, respectively, and the LF-specific factors explained 68.6% and 77.4% of the changes in M1 and M2 in China’s money market.

According to the BIC principle, it can be seen that the model obtained from the regression of crude oil prices tends to choose a model of common factor and HF-specific factor, and this conclusion is the same as that of the full sample.

The volatility of the crude oil price can be influenced by China’s macroeconomic (*ColumnHC*), but HF-specific factor accounts for the majority of its volatility (*ColumnH*). The adjusted *R*^2^ of the model in *ColumnC* serves as an example of this conclusion. These factors include the common factor and the HF-specific factor, which together account for 99.2% of the volatility of WTI crude oil price and 99.6% of the volatility of Brent crude oil price, respectively. Common factor and HF-specific factor together account for 99.7% of the volatility of the UAE Dubai crude oil price, 99.7% of the volatility in the Omani crude oil price. Similarly, for China’s largest trading partner, Indonesia, common factor and HF-specific factor account for 99.1% of the volatility of the Sinta crude oil price and 99.7% of the volatility in Minas crude oil price. Together, these two factors account for 99.7% of the price volatility of China Daqing crude oil. It is evident that the proportion of the fluctuations in the price of crude oil that are simultaneously explained by the common factor and the HF-specific factors is equivalent to what it was during the full sample.

**The COVID-19.** The regression results for the COVID-19 period, covering from January 2020 to December 2023, are covered in this section.

The proportion of changes in the SSE Composite Index that can be explained by the common factor and LF-specific factors exceeds 80%, and the proportion of changes in the SZSE Composite Index that can be explained by the common factor and LF-specific factors exceeds 82.1%, and unlike in the full sample period, most of the reasons for the changes in the SSE Composite Index are explained by the LF-specific factors instead of the common factor.

The regression results for the average A-share PE ratio are the same as those for the SSE Composite Index. At the same time, the proportion of explanation of LF-specific factors on the PE ratio of the single industry is lower than the average PE ratio of A-share, in which the influence on real estate industry is the largest, followed by basic chemical industry, computer, food and beverage. In other words, during the COVID-19 period, the proportion of factor explanation varies in the order of magnitude compared to the full sample period. Furthermore, the assessment level of the stock prices of these industries is more influenced by changes in China’s macroeconomic environment than by changes in the price of crude oil. Regression of the LF-specific factors on CPI has an adjusted *R*^2^ of 79.8%, and regression on PPI has an adjusted *R*^2^ of 92.9%. The adjusted *R*^2^ values obtained from regressions of the export price index and import price index are 82.1% and 86.8%, respectively. The LF-specific factors account for 84.1% and 93.8% of the changes in M1 and M2 in China’s money market.

It is evident from the BIC principle that the model derived from the regression of crude oil prices favors the model of common factor and HF-specific factor; this conclusion holds consistently for the full sample and the Financial Crisis. The adjusted *R*^2^ value of the *ColumnC* model illustrates this result. Whereas HF-specific factor (*ColumnH*) account for the majority of the volatility in crude oil prices, the China’s macroeconomic conditions have an impact as well (*ColumnHC*). The proportion of each crude oil price fluctuation that can be accounted for by the common factor and HF-specific factor surpasses 90%, as the table’s *ColumnHC* shows. This result is in line with findings from the full sample and the Financial Crisis.

#### 3.3.3 Plot of auto-correlation function of factors.

**Fig 2:**
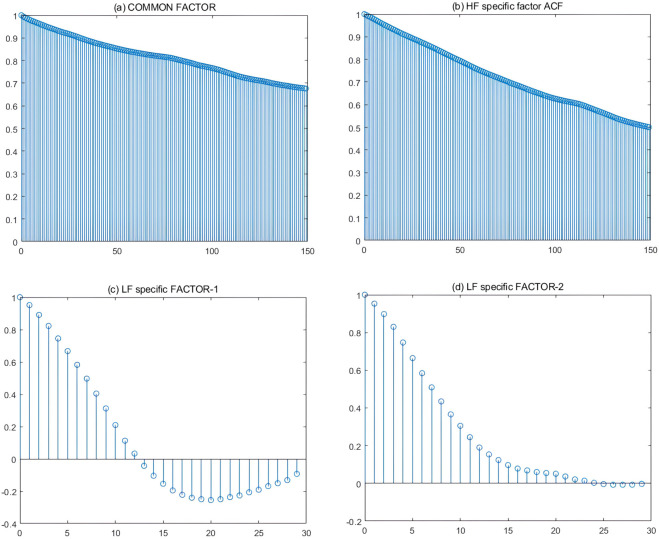
Estimated auto-correlation functions for common factor and group-specific factors (aggregation first). The auto-correlation function can be used to characterize the degree of correlation between factors at different moments. (*a*) shows a plot of the sample auto-correlation function of the common factor estimates at high frequency, (*b*) shows a plot of the sample auto-correlation function of the HF factor estimates at high frequency, (*c*) to (*d*) show a plot of the sample auto-correlation function of LF factors estimate at low frequency. Factor values are estimated from 60 China’s macroeconomic indicators (LF data) and 11 crude oil prices (HF data) using a mixed-frequency group factor model with $k^{C}=k^{H}=1$ and *k*^*L*^ = 2. The sample period is 200501–202403.

The autocorrelation functions of the group-specific factors and common factor, which were computed over the full sample period, are plotted in Fig 2. Fig 2 illustrates that during the observation period (6 months plus), the autocorrelations of the common factors and HF-specific factors are strong and positive, and they declined less. The first LF-specific factor exhibits a change in autocorrelation from positive to negative in the 20th period (20th months), with the negative auto-correlation gradually increasing. Meanwhile, the second LF-specific factor exhibits a positive auto-correlation in the 11th period (11st months), with a larger decrease in the positive auto-correlation. As a result, the common factor and group-specific factors exhibit time-series dependence; that is, the correlation between the fluctuations in crude oil prices and the China’s macro-economy is long-term and time-series dependent.

Plots of the estimated autocorrelation functions for the common factor and group-specific factors in the Financial Crisis and the COVID-19 are shown in the Appendix.

## 4 Conclusions

This study employs a mixed-frequency group factor model to examine the correlation between China’s macroeconomic market and the crude oil market from January 2005 to March 2024, addressing the frequency mismatch between 11 daily crude oil price indicators (HF) and 60 monthly Chinese macroeconomic indicators (LF) by decomposing volatility drivers into common factors (cross-market spillovers) and group-specific factors (LF-specific for macro, HF-specific for crude oil).

By decomposing volatility drivers into common factors (capturing cross-market spillovers) and group-specific factors (low-frequency, LF-specific, for macro; high-frequency, HF-specific, for crude oil), and validating results via adjusted coefficient of determination (*R*^2^) and Bayesian Information Criterion (BIC) model selection, we derive three core findings.

First, the two markets exhibit mutual but highly asymmetric influence: Common factors explain approximately 46% of China’s macroeconomic volatility (full sample), with key sectors (e.g., financial markets, industrial prices, monetary supply) highly sensitive to crude oil shocks; in contrast, common factors account for less than 10% of crude oil price volatility, as HF-specific factors (e.g., OPEC+ production adjustments, EIA inventory reports) dominate oil price fluctuations (explaining 93.8% of volatility alone).

Second, the correlation is time-varying, with crises weakening cross-market spillovers: During the 2007–2009 Financial Crisis and 2020–2023 COVID-19 period, LF-specific factors (e.g., China’s 4-trillion-yuan stimulus, post-pandemic consumption vouchers) explain 79.6%–86.1% of macroeconomic volatility, and BIC favors “LF-specific only” models for 68.3%–70.0% of macro indicators—reflecting the decoupling of China’s macroeconomy from crude oil via domestic policies. The crude oil market, however, retains global-driven volatility: the “ommon + HF-specific” model remains BIC-preferred for 99.9% of oil prices, with combined explanatory power exceeding 99%.

Third, factors exhibit long-term persistence: Autocorrelation analysis shows common and group-specific factors maintain strong positive autocorrelation for 6+ months, indicating lasting spillover effects (e.g., oil-driven inflation persists in China’s PPI for 3–6 months).

These findings confirm crude oil as a key external constraint on China’s macroeconomic stability, while China’s macro conditions have limited impact on global oil pricing (consistent with its status as a “price taker”).

Based on the conclusions drawn from this study, we can provide some policy recommendations from three aspects.

Firstly, as macroeconomic policymakers: (1) For the hedge short-term shocks, they can strengthen strategic crude oil reserves, to expand strategic reserves from the current 90 days of net imports to 120 days, and establish a “dynamic adjustment mechanism” (e.g., increase reserves when Brent crude falls below $70/barrel, release when it exceeds $120/barrel), to mitigate oil shocks and stabilize growth. (2) In the normal periods, they can integrate oil price signals into monetary policy (e.g., use interest rate hikes to offset oil-driven inflation when PPI exceeds 3%, and RRR cuts to support growth when oil prices fall sharply). In the crisis periods, they can prioritize domestic tools (e.g., 2020-style consumption vouchers, targeted credit support for manufacturing) to avoid over-reliance on global oil market stability. That is, macroeconomic policymakers can adopt “Dual-Track Policy Matching” for Normal vs. Crisis Periods.

Secondly, for sensitive industries, they should reduce oil dependence and manage risks. (1) Promote energy substitution in high-oil-consumption sectors. In manufacturing, they should subsidize natural gas use in chemical/steel plants (replace 20% of oil-derived feedstocks by 2030). In transportation, they should expand electric vehicle (EV) charging infrastructure, target 50% of new commercial vehicles (trucks, buses) to be EVs by 2027. (2) Strengthen Risk Management in the Financial Sector. In banks, they should cap loans to high-energy-consuming industries (e.g., oil refining) at 15% of total corporate loans; require stress tests for oil price swings (e.g., simulate a 50% oil price hike). In stock exchanges, they should Launch oil-linked financial products to help investors hedge energy-related portfolio risks.

Thirdly, as market participants, they should optimize investment and trading strategies. (1) As crude oil traders, they should focus on HF-Specific Factors. For short-term trading, Monitor EIA weekly inventory reports (released Wednesdays) and OPEC+ ministerial meetings—these drive 70% of daily oil price volatility (consistent with HF-specific factors’ 93.8% explanatory power). For long-term positions, using Chinese macro indicators (e.g., M1 growth >8%, secondary industry investment >5%) judges oil demand trends, as these signal increased manufacturing oil consumption. (2) As Equity Investors, they should adjust sector allocation based on oil shocks. For oil price surges, they can consider to underweight energy-intensive sectors (e.g., chemicals, aviation) and overweight oil-independent sectors (e.g., consumer services, healthcare). For oil price declines, they can consider to overweight refineries and transportation sectors, as the profit margins expand with lower input costs.

This study focuses on the “correlation” between the two markets but does not explore “predictability”and “usal inference”—future research could use the mixed-frequency group model to test whether China’s macro indicators (e.g., PMI) can forecast crude oil volatility, or vice versa. Additionally, expanding the sample to include other energy markets (e.g., natural gas, coal) could clarify the substitution effect of energy sources on the macro-oil linkage. Overall, our findings confirm that crude oil remains a key “external constraint” on China’s macroeconomic stability. By combining targeted reserves management, flexible policy matching, and industry-level energy transformation, China can better mitigate oil market risks and achieve sustainable economic growth.

**Table 5:** Abbreviations for macroeconomic variables in China.

Macroeconomic Indicators in China			
**Level1**	**Level2**	**Variable Name**	**Abbreviation**
Finance and Credit	Financial Market	SH composite index	SSE
Finance and Credit	Financial Market	SZ composite index	SZSE
Finance and Credit	Financial Market	A share:Average PE ratio	PE
Finance and Credit	Financial Market	PE-Basic chemical	PE-BCE
Finance and Credit	Financial Market	PE-automotive	PE-Auto
Finance and Credit	Financial Market	PE-food and beverage	PE-FB
Finance and Credit	Financial Market	PE-medical biology	PE-MB
Finance and Credit	Financial Market	PE-real estate	PE-RE
Finance and Credit	Financial Market	PE-computer	PE-C
Finance and Credit	Financial Market	Excess reserve	ER
Finance and Credit	Financial Market	Personal housing provident fund loan interest rate	PHL-5+
Finance and Credit	Money Market	M2	M2
Finance and Credit	Money Market	M1	M1
Finance and Credit	Money Market	M2:year-on-year	M2-YoY
Finance and Credit	Money Market	M1:year-on-year	M1-YoY
Finance and Credit	Money Market	reserve currency (base currency)	RC
Finance and Credit	Money Market	Money multiplier	MM
Finance and Credit	Money Market	Net cash disbursements: value for the month	NCI
Finance and Credit	Money Market	RMB Deposit Reserve Ratio-large	DRR-L
Finance and Credit	Money Market	RMB deposit reserve ratio-small and medium-sized	DRR-M/S
Finance and Credit	Money Market	Size of social financing: monthly value	SF-M
Finance and Credit	Money Market	Social Financing Scale:New RMB Loans:monthly value	SFS-NL
Finance and Credit	Money Market	Financial Institutions: New RMB Loans: Current Month’s Value	FI-NL
Finance and Credit	Money Market	Financial institutions: Deposit balances: RMB	FI-DB
Finance and Credit	Money Market	Financial institutions: balances of foreign currency deposits	FI-FD
Overall National Economy	Price Levels	CPI	CPI
Overall National Economy	Price Levels	RPI	RPI
Overall National Economy	Price Levels	PPI	PPI
Overall National Economy	Prosperity	Consumer Confidence Index	CCI
Overall National Economy	Prosperity	Manufacturing PMI	PMI-M
Overall National Economy	Prosperity	OECD Comprehensive Leading Indicators:China	OECD
Overall National Economy	Prosperity	Citi China Economic Surprise Index:Month:Average	CCESI
Growth Momentum	Investment	Fixed Asset Investment Completion: Cumulative Year-on-Year	FAIC
Growth Momentum	Investment	Percentage of investment in fixed assets: secondary sector	FAIP
Growth Momentum	Investment	Fixed Asset Investment Completion:New Construction:Cumulative Year-on-Year	FAIC-N
Growth Momentum	nvestment	China Housing Prosperity Index (CHPI)	CHPI
Growth Momentum	Investment	Fixed Asset Investment Completion:Real Estate:Cumulative Y/Y	FAIC-RE
Growth Momentum	Investment	Fixed Asset Investment Completion:Manufacturing:Cumulative Y/Y	FAIC-M
Growth Momentum	Investment	Fixed Asset Investment Completion:Infrastructure Investment:Cumulative Y/Y	FAIC-II
Growth Momentum	Consumption	Total retail sales of consumer goods: month-over-month	CG-MoM
Growth Momentum	Consumption	Total retail sales of consumer goods: current month’s value	CG-CM
Growth Momentum	Consumption	Movie box office receipts: current month’s value	MBOR
Growth Momentum	External Trade	Official reserve assets: foreign exchange reserves	ORA-FER
Growth Momentum	External Trade	Surplus of foreign exchange settlement between banks and clients: current month’s value	BCS
Growth Momentum	External Trade	Official reserve assets: gold (amount of pure gold in ounces)	ORA-G
Growth Momentum	External Trade	Export Price Index	EPI
Growth Momentum	External Trade	Import Price Index	IPI
Growth Momentum	External Trade	Export quantity index	EQI
Growth Momentum	External Trade	Import quantity index	IQI
Government Finance	Taxes and Finance	Public finance revenue: month-on-month	PFR
Government Finance	Taxes and Finance	Public fiscal expenditure	PFE
Government Finance	Taxes and Finance	Fiscal balance:current month’s value	FB
Government Finance	Taxes and Finance	Financial institutions:Balance of financial deposits:RMB	FDB
Government Finance	Taxes and Finance	Tax revenues:Domestic value added tax: current month’s value	DVAT-CMV
Government Finance	Taxes and Finance	Tax revenues:Domestic VAT:Month-over-month	DVAT-MoM
Government Finance	Taxes and Finance	Tax revenues:Individual income tax:Current month’s value	IIT-CMV
Government Finance	Taxes and Finance	Tax revenues:Individual income tax:Month-over-month	IIT-MoM
Government Finance	Taxes and Finance	Tax revenues:Corporate income tax:Current month’s value	CIT-CMV
Government Finance	Taxes and Finance	Tax revenues:Corporate income tax:Month-over-month	CIT-MoM
Government Finance	Taxes and Finance	Tax revenues:tariffs:month-on-month	Tariffs-MoM

## A1 Variable information

### A1.1 Abbreviations for macroeconomic variables

In our work, we have chosen 11 crude oil daily frequency current prices and 60 the China’s macroeconomic monthly frequency indicators, which cover four components: Finance and Credit, Overall National Economy, Growth Momentum, Government Finance. Among them, Financial and Credit includes the financial market and the money market, the money market includes M1, M2 and so on, while the financial market includes the SSE Composite Index and the SZSE Composite Index; Price level and prosperity are two aspects of the Overall National Economy, price level includes indicators like CPI, RPI, and PPI, while prosperity is measured by the manufacturing PMI, consumer confidence index, and other indicators; The three main drivers of Growth Momentum are investment, consumption, and external trade. For investment, indicators include total retail sales of consumer goods: month-over-month, and so on, for external trade, indicators include official reserve assets: gold, export price index, import price index and so on; Taxation and finance is example of Government Finance. Indicators related to taxes and finance include public finance revenue: month-on-month, public fiscal expenditure, and so on, which are shown on the Table 5.

### A1.2 Statistics of all variables

The statistical values for each variable are showed in Table 6 prior to any treatment being applied. The table displays the following statistics in order from left to right: mean, standard deviation, minimum, lower quartile, median, upper quartile, maximum, skewness, and kurtosis.

## A2 Factor auto-correlation function plot

This section shows the autocorrelation function graphs based on the factors estimated from each of the two sub-samples.

### A2.1 The financial crisis

**Fig 3:**
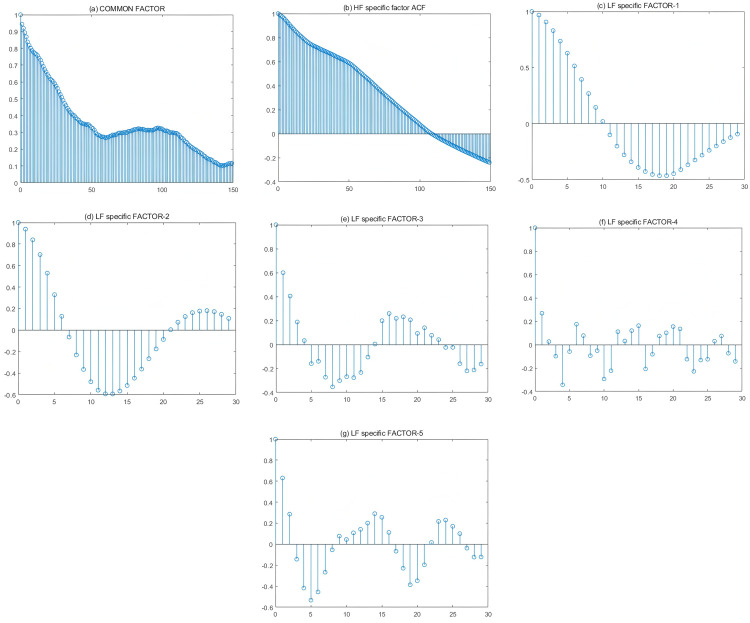
Estimated auto-correlation functions for common and group-specific factors (aggregation first).

The auto-correlation functions can be used to characterize the degree of correlation between factors at different moments. In Fig 3, (*a*) shows a plot of the sample auto-correlation function for the common factor estimates at high frequency. (*b*) shows a plot of the sample auto-correlation function for the HF factor estimates at high frequency. (*c*) to (*g*) show plots of sample auto-correlation functions for the LF factor estimates at low frequency. Factor values are estimated from 60 China’s macroeconomic indicators (LF data) and 11 high-frequency crude oil prices (HF data) using a mixed-frequency group factor model with $k^{C}=k^{H}=1$ and *k*^*L*^ = 5. The sample period is 200701–200912.

Fig 3 are the plots of the autocorrelation functions of the common factor and group-specific factors estimated during the Financial Crisis period. From the Fig 3, We can get that the common factor has high and positive autocorrelations during the Financial Crisis (more than 6 months), and the HF-specific factor has high positive autocorrelations over a longer period of time (more than 4 months), which turns from positive to negative autocorrelations in around 4.7 months, with the correlation gradually increasing.

**Table 6:** Statistical values of all variables. The statistical values for each variable are displayed prior to any treatment being applied. The dataset is from January 2005 to March 2024.

name	mean	std	min	Q1	median	Q3	max	skew	kurt
SSE	2830.74	787.66	1042.18	2308.89	2947.85	3258.27	5230.02	0.12	1.07
SZSE	1406.36	618.11	246.20	960.33	1348.20	1935.16	2891.00	–0.08	–0.92
PE	18.42	9.70	9.70	13.04	15.82	19.13	68.00	2.77	8.37
PE-BCE	27.78	10.82	12.98	19.55	25.74	33.18	69.99	0.97	0.58
PE-Auto	22.76	9.46	9.04	15.48	20.34	28.39	65.30	1.28	2.07
PE-FB	34.86	13.56	17.11	26.74	31.60	38.87	94.02	1.73	3.69
PE-MB	38.42	11.68	21.30	31.09	37.38	42.44	92.39	1.68	4.61
PE-RE	21.50	16.04	7.19	11.81	16.24	23.99	112.85	2.60	8.15
PE-C	52.39	17.68	20.11	40.98	51.27	60.38	142.70	1.22	3.89
ER	0.70	0.22	0.35	0.72	0.72	0.72	1.62	0.20	1.49
PHL-5+	3.91	0.69	3.10	3.25	3.87	4.50	5.22	0.33	–1.38
M2	1313518.17	796165.85	257708.47	590624.01	1199236.31	1905282.26	2995572.97	0.43	–0.96
M1	376112.14	192002.45	92814.95	210019.47	332023.23	549630.72	695595.48	0.13	–1.37
M2-YoY	13.80	4.77	8.00	10.10	13.20	16.73	29.74	1.18	1.59
M1-YoY	11.42	7.99	–1.90	4.95	9.40	16.00	38.96	0.90	0.31
RC	233374.83	97819.87	56219.53	133540.55	276652.23	308507.30	389036.93	–0.57	–1.12
MM	5.28	1.33	3.61	4.20	4.79	6.22	8.22	0.82	–0.63
NCI	429.74	4077.80	–14900.00	–895.50	357.00	1359.00	18300.00	0.87	6.40
DRR-L	14.97	4.06	7.50	11.50	15.50	18.00	21.50	–0.24	–1.03
DRR-M/S	13.15	3.92	7.00	9.50	13.50	16.50	19.50	–0.10	–1.40
SF-M	15644.30	12026.49	–974.00	7430.00	12397.00	19886.96	65364.00	1.64	3.18
SFS-NL	9780.94	8260.41	–314.00	4815.50	7740.00	11870.15	49314.00	2.23	6.15
FI-NL	9880.97	7975.53	–321.00	5083.50	7718.00	12500.00	49200.00	2.18	6.33
FI-DB	1278164.95	775539.11	245368.63	589801.89	1124704.80	1864345.06	2906999.38	0.43	–0.97
FI-FD	5267.24	2947.59	1522.00	2063.00	5936.00	7789.50	10500.00	0.03	–1.51
CPI	2.34	1.89	–1.80	1.40	2.00	2.90	8.70	0.88	1.35
RPI	1.91	1.86	–2.50	0.80	1.65	2.67	8.10	0.89	1.46
PPI	1.48	4.61	–8.20	–2.15	1.69	5.39	13.50	0.11	–0.84
CCI	107.99	10.09	85.50	102.85	108.00	113.35	127.00	–0.24	–0.25
PMI-M	51.34	2.71	35.70	50.10	51.00	52.60	59.20	–0.90	7.02
OECD	99.92	1.62	88.77	99.51	100.19	100.68	103.57	–2.33	12.23
CCESI	5.57	48.02	–141.00	–19.74	6.99	33.06	143.25	–0.12	1.16
FAIC	16.02	10.79	–24.50	6.55	17.30	25.60	35.00	–0.55	0.37
FAIP	38.49	5.42	26.95	32.70	40.90	42.95	45.20	–0.63	–1.10
FAIC-N	18.12	12.07	–26.40	10.10	17.20	26.75	42.80	–0.28	0.64
CHPI	99.43	3.65	92.13	95.90	100.70	101.79	106.59	–0.33	–0.98
FAIC-RE	15.34	13.37	–18.10	5.85	14.00	27.12	37.60	–0.17	–0.95
FAIC-M	17.15	13.59	–31.50	6.40	17.00	29.05	39.20	–0.52	0.43
FAIC-II	16.07	11.91	–26.86	8.63	17.29	22.17	50.78	0.21	1.90
CG-MoM	11.53	7.18	–20.50	9.00	12.10	15.35	34.20	–1.01	4.57
CG-CM	22273.94	11601.14	4663.30	11330.35	22386.71	32579.10	43550.00	0.07	–1.31
MBOR	236886.45	228250.13	7289.80	46400.00	179700.00	362150.00	971391.29	1.40	1.90
ORA-FER	27510.84	8944.19	6236.46	23585.30	31123.28	32220.95	39932.13	–1.08	0.01
BCS	119.96	280.12	–1279.96	–39.28	125.26	291.00	925.72	–1.01	4.39
ORA-G	4346.10	1790.59	1929.00	3389.00	3389.00	6177.50	7258.00	–0.03	–1.58
EPI	102.44	5.39	90.30	99.10	102.10	106.30	115.70	–0.01	–0.29
IPI	102.91	9.42	79.60	97.35	102.00	110.40	122.70	–0.13	–0.47
EQI	109.46	16.05	76.10	99.95	108.20	117.40	244.60	2.72	21.15
IQI	106.84	11.58	63.70	99.60	107.10	112.65	163.50	0.54	3.04
PFR	12.37	15.63	–41.34	3.65	9.40	20.45	69.96	0.35	1.47
PFE	13.90	15.47	–19.90	4.48	11.60	21.72	84.92	1.05	2.75
FB	–2609.58	6102.29	–19795.60	–6739.80	–549.41	1676.67	10693.51	–0.95	0.37
FDB	37828.09	15149.50	7447.00	27874.83	38348.24	49075.50	70411.12	–0.18	–0.77
DVAT-CMV	2983.12	1659.06	–1555.00	1615.62	2489.72	4491.00	7689.07	0.37	–0.70
DVAT-MoM	15.82	50.58	–124.67	2.21	10.82	20.87	508.10	7.36	70.00
IIT-CMV	660.68	350.56	152.33	358.25	591.83	917.50	1768.61	0.47	–0.61
**name**	**mean**	**std**	**min**	**Q1**	**median**	**Q3**	**max**	**skew**	**kurt**
IIT-MoM	12.18	22.15	–51.26	1.31	13.97	22.42	81.35	–0.03	1.82
CIT-CMV	1956.54	1854.07	–737.38	620.94	1061.00	3086.31	6659.00	1.00	–0.24
CIT-MoM	16.37	40.76	–149.43	–2.94	9.91	27.31	245.48	2.02	10.46
Tariffs-MoM	6.70	21.58	–41.75	–7.70	3.70	18.97	81.20	0.71	1.25
**Crude Oil Price**									
**name**	**mean**	**std**	**min**	**Q1**	**median**	**Q3**	**max**	**skew**	**kurt**
WTI	71.68	21.77	7.30	54.02	70.18	88.84	137.21	0.27	–0.45
Brent	76.10	24.70	13.28	57.10	72.57	96.63	144.22	0.29	–0.80
UAE Dubai	73.75	23.88	13.56	55.05	70.62	93.64	140.77	0.26	–0.84
Omani	74.11	23.80	13.68	55.60	70.84	93.85	141.30	0.27	–0.81
Tapis	79.79	25.75	15.09	60.79	75.42	100.51	156.02	0.34	–0.73
Minas	74.84	25.88	11.67	55.39	70.37	95.89	149.76	0.41	–0.63
Duri	71.97	25.73	19.30	50.68	68.39	94.35	133.34	0.28	–0.99
Sinta	70.81	24.97	12.99	53.13	65.56	89.60	138.64	0.50	–0.64
China Daqing	72.54	25.67	12.56	53.33	68.59	93.82	143.75	0.33	–0.75
China Shengli	71.24	25.47	19.67	50.76	66.94	92.83	130.14	0.31	–0.94
OPEC-Pacage	73.94	24.48	12.22	54.41	70.91	95.14	140.73	0.24	–0.85

The first LF-specific factor shifts from a positive to a negative correlation at period 10 (10th month), and the negative correlation goes from strong to weak during the observation period. The second LF-specific factor shifts from a positive autocorrelation to a negative autocorrelation at period 7 (7th month) and from a negative auto-correlation to a positive auto-correlation at period 21 (21st month), and the correlations are all progressively stronger and then weaker. The other three LF-specific factors switch between positive and negative correlations more frequently and have more significant autocorrelations.

### A2.2 The COVID-19 period

**Fig 4:**
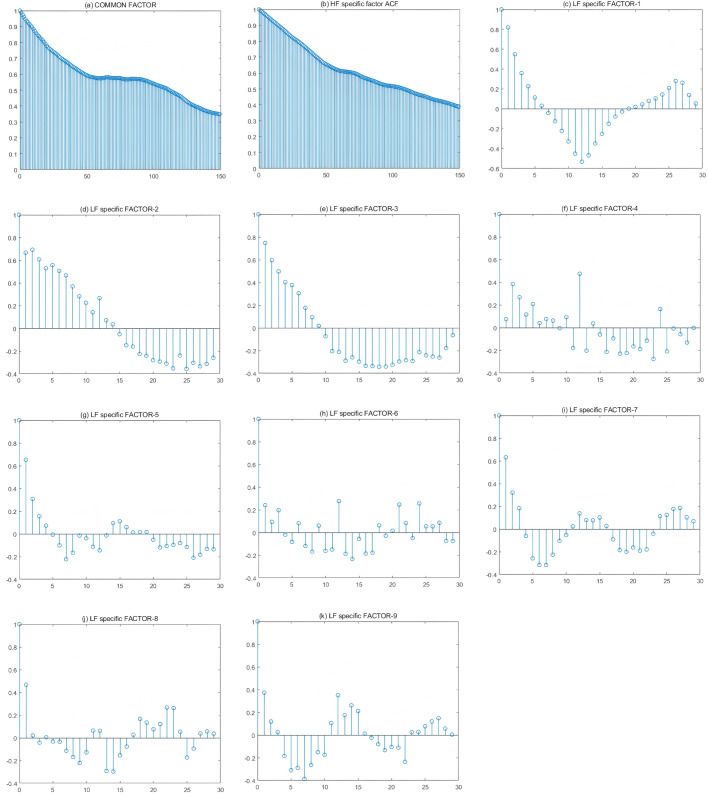
Estimated auto-correlation functions for common and group-specific factors (aggregation first).

Fig 4 in the appendix presents graphs of autocorrelation functions for common factor and group-specific factors estimated during the COVID-19 period. As can be seen in Fig 4, the common factor and the HF-specific factor have high and positive autocorrelations during the observation period (more than 6 months).

The first LF-specific factor has a gradually weakening auto-correlation in the first 6 periods, turns from a positive to a negative correlation in period 7 (7th month), with the correlation gradually increasing and then decreasing, and then the auto-correlation turns to a positive auto-correlation again in period 20, with a gradually increasing and then decreasing auto-correlation (during the observation period). The second LF-specific factor has a decreasing auto-correlation in the first 14 periods, and then the auto-correlation changed from positive to negative in the 15th period (15th month), and the auto-correlation gradually increased and then slowly decreased (during the observation period). The third LF-specific factor has a decreasing auto-correlation in the first 9 periods, and the auto-correlation changed from positive to negative in the 10th period (10th month), and the auto-correlation is high and fluctuated slowly (during the observation period). The other six LF-specific factors have frequent switching of positive and negative correlations and more pronounced autocorrelations.

## A3 Regression results

The regression results for the full sample (200501-202403) and two sub-samples (200701-200912 and 202001-202312) are presented in Tables 7 and 8. All results are for common factor and group-specific factors estimated from a data panel of 11 crude oil price indicators and 60 China’s macroeconomic indicators.

**Table 7:** Regression results of China’s macroeconomic indicators on estimated factors. “L” is the ${\bar{R}}^{2}$ value obtained by regressing China’s macroeconomic variables on low-frequency specific factors. “C” is the ${\bar{R}}^{2}$ value obtained by regressing China’s macroeconomic variables on common factors. “LC” is the ${\bar{R}}^{2}$ value obtained by regressing China’s macroeconomic variables on low-frequency specific factors and common factors. “LC-C” is the difference between the ${\bar{R}}^{2}$ value of “LC” and “C”. “BIC” is the model preference results obtained based on BIC criteria.

Panel A	${\bar{R}}^{2}$														
Macro	**200501-202403**					**200701-200912**					**202001-202312**				
**Name**	**L**	**LC**	**C**	**LC-C**	**BIC**	**L**	**LC**	**C**	**LC-C**	**BIC**	**L**	**LC**	**C**	**LC-C**	**BIC**
	**(1)**	**(2)**	**(3)**	**(2)-(3)**		**(1)**	**(2)**	**(3)**	**(2)-(3)**		**(1)**	**(2)**	**(3)**	**(2)-(3)**	
SSE	0.843	0.867	–0.002	0.869	CL	0.844	0.894	0.29	0.604	CL	0.842	0.839	0.095	0.744	L
SZSE	0.471	0.878	0.279	0.599	CL	0.677	0.735	0.199	0.536	CL	0.821	0.816	0.087	0.729	L
PE	0.312	0.625	0.324	0.301	CL	0.926	0.944	0.241	0.703	CL	0.943	0.942	0.352	0.590	L
PE-BCE	0.356	0.365	0.039	0.326	L	0.937	0.935	0.007	0.928	L	0.893	0.892	0.496	0.396	L
PE-Auto	0.634	0.647	0.042	0.605	CL	0.926	0.930	–0.015	0.945	L	0.525	0.515	0.105	0.41	L
PE-FB	0.614	0.721	0.111	0.610	CL	0.953	0.959	0.209	0.750	CL	0.889	0.889	0.228	0.660	L
PE-MB	0.437	0.489	0.088	0.401	CL	0.936	0.939	0.097	0.842	L	0.859	0.885	0.421	0.464	L
PE-RE	0.181	0.625	0.488	0.137	CL	0.856	0.893	0.124	0.769	CL	0.921	0.919	0.288	0.631	L
PE-C	0.528	0.652	0.047	0.605	CL	0.927	0.929	0.004	0.925	L	0.876	0.886	0.298	0.589	L
ER	0.110	0.502	0.306	0.195	CL	0.852	0.847	0.262	0.585	L	0.613	0.604	0.014	0.590	L
PHL-5+	0.071	0.591	0.431	0.160	CL	0.905	0.911	0.378	0.533	L	0.780	0.785	0.100	0.685	L
M2	0.130	0.651	0.423	0.227	CL	0.774	0.775	0.116	0.659	L	0.938	0.949	0.286	0.663	L
M1	0.128	0.707	0.484	0.224	CL	0.686	0.693	0.047	0.646	L	0.841	0.867	0.229	0.638	L
M2-YoY	0.036	0.463	0.410	0.053	CL	0.867	0.872	0.086	0.786	L	0.638	0.628	0.308	0.320	L
M1-YoY	0.036	0.272	0.244	0.028	C	0.886	0.895	–0.023	0.917	L	0.439	0.450	0.048	0.402	L
RC	0.135	0.727	0.469	0.258	CL	0.627	0.621	0.055	0.565	L	0.794	0.802	0.180	0.622	L
MM	0.123	0.428	0.264	0.164	CL	0.684	0.673	–0.009	0.682	L	0.853	0.856	0.285	0.571	L
NCI	–0.007	–0.011	–0.004	–0.007	C	–0.028	–0.062	–0.029	–0.033	C	0.210	0.224	–0.013	0.238	L
DRR-L	0.119	0.118	–0.004	0.122	L	0.616	0.621	–0.027	0.648	L	0.924	0.932	0.353	0.579	L
DRR-M/S	0.087	0.100	0.020	0.080	L	0.643	0.649	0.003	0.646	L	0.917	0.920	0.326	0.593	L
SF-M	0.067	0.312	0.199	0.113	CL	0.297	0.275	0.136	0.139	C	0.928	0.926	–0.018	0.944	L
SFS-NL	0.050	0.269	0.178	0.091	CL	0.411	0.396	0.142	0.255	C	0.981	0.981	–0.013	0.995	L
FI-NL	0.056	0.304	0.202	0.103	CL	0.412	0.397	0.141	0.256	C	0.973	0.973	–0.011	0.984	L
FI-DB	0.133	0.654	0.425	0.229	CL	0.799	0.801	0.123	0.678	L	0.936	0.946	0.277	0.670	L
FI-FD	0.116	0.737	0.517	0.219	CL	0.950	0.949	0.258	0.691	L	0.920	0.922	–0.022	0.944	L
CPI	0.219	0.401	0.148	0.253	CL	0.943	0.943	0.369	0.574	L	0.798	0.795	–0.014	0.809	L
RPI	0.351	0.463	0.081	0.382	CL	0.952	0.952	0.336	0.615	L	0.588	0.587	0.127	0.459	L
PPI	0.717	0.737	–0.003	0.741	CL	0.982	0.982	0.277	0.705	L	0.929	0.934	–0.008	0.942	L
CCI	0.277	0.372	0.138	0.234	CL	0.886	0.883	0.288	0.595	L	0.956	0.965	0.472	0.493	L
PMI-M	0.1	0.279	0.144	0.135	CL	0.741	0.733	0.009	0.725	L	0.440	0.433	0.001	0.433	L
OECD	0.089	0.109	0.024	0.085	L	0.933	0.931	0.061	0.870	L	0.959	0.960	0.001	0.959	L
CCESI	0.172	0.247	0.056	0.192	CL	0.833	0.880	0.156	0.724	CL	0.867	0.866	–0.019	0.885	L
FAIC	0.109	0.519	0.324	0.195	CL	0.792	0.798	0.118	0.680	L	0.960	0.959	–0.021	0.980	L
FAIP	0.120	0.617	0.451	0.166	CL	0.392	0.378	–0.029	0.407	L	0.667	0.679	0.255	0.424	L
FAIC-N	0.191	0.532	0.264	0.269	CL	0.763	0.763	0.164	0.599	L	0.962	0.962	0.041	0.92	L
CHPI	0.559	0.639	0.031	0.608	CL	0.912	0.925	0.410	0.515	CL	0.970	0.969	0.511	0.458	L
FAIC-RE	0.212	0.545	0.230	0.315	CL	0.835	0.830	0.268	0.562	L	0.952	0.952	0.169	0.783	L
FAIC-M	0.136	0.557	0.319	0.238	CL	0.724	0.725	0.162	0.563	L	0.956	0.958	–0.002	0.960	L
FAIC-II	0.137	0.307	0.168	0.138	CL	0.943	0.944	0.238	0.705	L	0.954	0.954	0.034	0.919	L
CG-MoM	0.039	0.218	0.140	0.078	CL	0.762	0.755	0.112	0.643	L	0.93	0.928	0.007	0.921	L
CG-CM	0.120	0.724	0.499	0.225	CL	0.559	0.564	0.015	0.549	L	0.638	0.634	–0.003	0.637	L
MBOR	0.043	0.414	0.312	0.102	CL	0.290	0.277	0.013	0.265	C	0.541	0.563	–0.022	0.585	L
ORA-FER	0.176	0.566	0.286	0.281	CL	0.665	0.670	0.049	0.621	L	0.626	0.619	0.159	0.460	L
BCS	0.221	0.378	0.099	0.279	CL	0.673	0.669	0.294	0.375	L	0.397	0.393	0.121	0.272	L
ORA-G	0.133	0.747	0.507	0.240	CL	0.800	0.795	0.112	0.682	L	0.850	0.858	0.006	0.852	L
EPI	0.266	0.324	0.029	0.294	CL	0.941	0.947	0.177	0.770	CL	0.821	0.820	0.186	0.634	L
IPI	0.651	0.680	0.002	0.678	CL	0.976	0.975	0.295	0.680	L	0.868	0.870	0.005	0.865	L
EQI	0.233	0.259	0.005	0.254	CL	0.663	0.661	0.173	0.488	L	0.784	0.784	0.035	0.749	L
IQI	0.141	0.196	0.031	0.165	CL	0.843	0.838	0.083	0.755	L	0.597	0.586	0.244	0.342	L
PFR	0.083	0.288	0.177	0.111	CL	0.747	0.738	0.115	0.624	L	0.864	0.863	–0.021	0.884	L
PFE	–0.004	0.145	0.133	0.012	C	0.058	0.034	–0.029	0.063	C	0.011	0.062	0.031	0.031	L
FB	–0.002	0.118	0.111	0.007	C	–0.101	–0.09	–0.001	–0.089	C	0.705	0.710	–0.007	0.717	L
FDB	0.140	0.640	0.402	0.238	CL	0.568	0.639	–0.010	0.649	CL	0.592	0.583	0.067	0.516	L
DVAT-CMV	0.109	0.584	0.408	0.177	CL	0.072	0.065	–0.022	0.087	C	0.513	0.509	0.019	0.490	L
DVAT-MoM	0.017	0.021	0.007	0.014	C	0.835	0.835	0.381	0.455	L	0.957	0.956	–0.016	0.972	L
IIT-CMV	0.139	0.606	0.382	0.224	CL	0.024	–0.010	–0.029	0.020	C	0.428	0.425	–0.022	0.446	L
IIT-MoM	0.071	0.109	0.038	0.071	CL	0.598	0.591	0.160	0.431	L	0.787	0.830	0.167	0.663	L
CIT-CMV	0.032	0.116	0.064	0.053	CL	–0.122	–0.136	–0.018	–0.118	C	0.921	0.919	–0.021	0.941	L
CIT-MoM	0.020	0.049	0.018	0.031	C	0.943	0.944	–0.013	0.957	L	0.392	0.380	–0.022	0.401	L
Tariffs-MoM	0.314	0.424	0.075	0.349	CL	0.946	0.949	0.417	0.532	L	0.305	0.294	–0.019	0.313	L

**Table 8:** Regression results of crude oil prices on the estimated factors. “H” is the ${\bar{R}}^{2}$ value obtained by regressing crude oil price variables on high-frequency specific factors. “C” is the ${\bar{R}}^{2}$ value obtained by regressing crude oil price variables on common factors. “HC” is the ${\bar{R}}^{2}$ value obtained by regressing China’s macroeconomic variables on high-frequency specific factors and common factors. “HC-C” is the difference between the ${\bar{R}}^{2}$ value of “HC” and “C”. “BIC” is the model preference results obtained based on BIC criteria.

Panel B	${\bar{R}}^{2}$														
**Crude oil**	**200501-202403**					**200701-200912**					**202001-202312**				
**Name**	**H**	**HC**	**C**	**HC-C**	**BIC**	**H**	**HC**	**C**	**HC-C**	**BIC**	**H**	**HC**	**C**	**HC-C**	**BIC**
	**(1)**	**(2)**	**(3)**	**(2)-(3)**		**(1)**	**(2)**	**(3)**	**(2)-(3)**		**(1)**	**(2)**	**(3)**	**(2)-(3)**	
WTI	0.843	0.956	0.095	0.861	CH	0.964	0.992	0.201	0.791	CH	0.988	0.990	0.234	0.756	CH
Brent	0.938	0.993	0.043	0.951	CH	0.982	0.996	0.164	0.832	CH	0.991	0.994	0.251	0.743	CH
UAE Dubai	0.952	0.995	0.032	0.963	CH	0.994	0.997	0.121	0.876	CH	0.992	0.996	0.258	0.738	CH
Omani	0.950	0.995	0.034	0.961	CH	0.993	0.997	0.124	0.873	CH	0.992	0.996	0.258	0.738	CH
Tapis	0.923	0.994	0.056	0.937	CH	0.98	0.994	0.163	0.831	CH	0.988	0.996	0.281	0.715	CH
Minas	0.886	0.992	0.089	0.903	CH	0.986	0.997	0.152	0.844	CH	0.996	0.997	0.184	0.813	CH
Duri	0.995	0.996	0.001	0.996	CH	0.997	0.999	0.066	0.933	CH	0.990	0.997	0.273	0.724	CH
Sinta	0.832	0.974	0.123	0.851	CH	0.985	0.991	0.135	0.856	CH	0.931	0.993	0.045	0.948	CH
Daqing	0.921	0.994	0.059	0.935	CH	0.985	0.997	0.158	0.839	CH	0.989	0.996	0.273	0.723	CH
Shengli	0.990	0.996	0.003	0.994	CH	0.997	0.998	0.067	0.931	CH	0.990	0.997	0.272	0.725	CH
OPEC-Pacage	0.956	0.996	0.029	0.967	CH	0.991	0.999	0.142	0.856	CH	0.992	0.997	0.265	0.733	CH
